# Facile One-Pot Conversion of (poly)phenols to Diverse (hetero)aryl Compounds by Suzuki Coupling Reaction: A Modified Approach for the Synthesis of Coumarin- and Equol-Based Compounds as Potential Antioxidants

**DOI:** 10.3390/antiox13101198

**Published:** 2024-10-03

**Authors:** Muthipeedika Nibin Joy, Igor S. Kovalev, Olga V. Shabunina, Sougata Santra, Grigory V. Zyryanov

**Affiliations:** 1 Ural Federal University named after the first President of Russia B. N. Yeltsin, 19 Mira Street, Yekaterinburg 620002, Russia; ekls85@yandex.ru (I.S.K.); shabunina.ov@talantiuspeh.ru (O.V.S.); sougatasantra85@gmail.com (S.S.); g.v.zyrianov@urfu.ru (G.V.Z.); 2 I. Ya. Postovskiy Institute of Organic Synthesis, Ural Division of the Russian Academy of Sciences, 22 S. Kovalevskoy Street, Yekaterinburg 620219, Russia

**Keywords:** coumarin, aryl imidazylates, Suzuki coupling, antioxidants, DPPH

## Abstract

A series of 16 (hetero)aryl compounds based on coumarin and equol has been efficiently synthesized by exploring the palladium-catalyzed Suzuki cross-coupling reactions. Polyphenol based on coumarin (4-methyl-7-hydroxy coumarin) was initially converted to corresponding coumarin imidazylate and then subjected to Suzuki coupling reaction with 4-methoxyphenylboronic acid to obtain the coupled product. This modified approach was later developed into a one-pot methodology by directly reacting the polyphenol with 1,1-sulfonyldiimidazole (SDI) and boronic acid in situ to obtain the Suzuki coupled product in one step. Moreover, an array of (poly)phenols based on coumarin and equol were later converted to diverse (hetero)aryl compounds by this optimized step-economic protocol. The synthesized compounds were then subjected to the screening of their potential antioxidant activities by 2,2-diphenyl-1-picrylhydrazyl (DPPH) assay. In our investigation, the compounds **4ah**, **4eh**, **4gh** and **4hh** exhibited promising antioxidant potential when compared to the reference standard, butylated hydroxytoluene (BHT). Structure activity relationship (SAR) studies revealed the importance of the presence of electron-donating substituents in enhancing the antioxidant activity of the synthesized compounds.

## 1. Introduction

The presence of highly reactive free radicals and oxygen species in the human body can cause numerous degenerative diseases [1,2,3] such as aging, atherosclerosis, cancer etc. One important reason for this issue is the fact that free radicals can abstract hydrogen atoms from membranes, lipids or DNA existing in biological systems [4]. In this scenario, the significance of eliminating free radicals from biological system has emerged as an urgent need. Accordingly, the sustainability of cellular machinery can be maintained, along with the prevention of oxidative diseases [5]. Free radicals can be effectively trapped and removed by providing antioxidants or free radical scavengers. Hence, oxidative damage can be mitigated by proper supplementation of compounds/molecules with excellent antioxidant properties [6,7]. A variety of natural and synthetic antioxidants has been reported so far and, among them, polyphenols are of utmost importance. Although most of the polyphenols are either extracted from plants or vegetable oils [8], they can also be synthesized in the laboratory on a large scale for various uses. Among the various types of available natural and synthetic polyphenols, coumarin-, equol- and daidzein-based compounds are highly important, owing to their varied applications in medicinal chemistry [9,10].

Coumarins, an important privileged class of benzopyrones, are reported to exhibit a wide spectrum of biological activities, including antimicrobial, antioxidant, anti-inflammatory, anti-tubercular and anticancer properties [11,12,13,14]. Coumarins are natural products found in green plants existing in a free or combined state. However, coumarins are also synthesized in the laboratory and their pharmacological significance is underlined by their presence in some important pharmaceutical drugs available in the market, such as warfarin (anti-coagulant), acenocoumarol (anti-coagulant), carbochromen (vasodilator), novobiocin (antibiotic), clorobiocin (antibiotic) and coumermycin A1 (antibiotic) [15,16,17,18]. Equol, an isoflavonoid belonging to polyphenols, is reported to display a wide range of pharmacological properties such as anti-androgenic, antioxidant and anti-inflammatory activities [19]. Equol is metabolized from daidzein in living organisms by intestinal bacteria. However, equol is also synthesized in laboratories in racemic form and as separate enantiomers [20].

In the modern arena of drug discovery, the role of palladium-catalyzed cross-coupling reactions, especially Suzuki coupling reactions, is highly significant [21,22,23]. A wide variety of heterocyclic architectures constituting diverse functional groups can be efficiently synthesized by Suzuki coupling reactions. Recently, we reported the utilization of aryl fluorosulfates as an efficient electrophilic coupling partner in Suzuki coupling for the synthesis of an array of coumarin derivatives [24]. However, our methodology required the initial conversion of phenols to a fluorosulfate-leaving group before subjecting it to Suzuki coupling reactions. This observation prompted us to develop a one-pot methodology for converting phenols directly to a leaving group in situ and react with Suzuki coupling conditions to obtain the desired biaryls as products in one step. In our successful trials, the (poly)phenols based on these natural products were directly converted to various (hetero)aryl compounds by a one-pot synthetic protocol. As a continuation of our ongoing research in the synthesis of biologically active molecules [25,26,27], we herein report a modified approach for the synthesis of a variety of compounds based on coumarin and equol. The antioxidant potential of these compounds was then evaluated by 2,2-diphenyl-1-picrylhydrazyl (DPPH) assay by considering the fact that it does not have to be generated prior to analysis. The structure activity relationship (SAR) studies of these compounds were also carried out at the later stage to get insights about the structural specificity and potency. 

## 2. Materials and Methods

### 2.1. General Information

All chemicals were purchased from commercial suppliers and used as delivered. Palladium catalysts and sulfonyldiimidazole (SDI) was procured from Sigma Aldrich, Beijing, China. DMF (Finar AR dry grade) was used directly for all the procedures. ^1^H NMR (400 or 600 MHz) and ^13^C NMR (100 or 150 MHz) spectra were recorded on Bruker Avance II and Bruker Avance NEO spectrometer (Bruker, Billerica, MA, USA), respectively. Chemical shifts are reported in parts per million (ppm) and coupling constants in Hertz (Hz). Tetramethylsilane (TMS) (δ = 0.00 ppm) or residual solvent peak in DMSO-*d_6_* (δ = 2.50 ppm) and CDCl_3_ (δ = 7.26 ppm) served as the internal standard for recording [28]. Molecular weights of unknown compounds were determined by Shimadzu GCMS-QP2010 Ultra gas chromatograph operating at an ionization potential of 70 eV (EI), (Shimadzu, Kyoto, Japan). Microanalyses were performed on PerkinElmer Series II CHNS/O 2400 elemental analyzer (PerkinElmer, Waltham, MA, USA). Melting points were determined using a Stuart SMP 3 apparatus. Thin-layer chromatography (TLC) was performed using Merck silica gel 60 F254 TLC plates (Merck, Darmstadt, Germany).

### 2.2. Procedure for the Synthesis of Coumarin Imidazylate Intermediate **2a**

In a sealed tube with screw cap, 4-methyl-7-hydroxy coumarin **1a** (1 mmol, 1 equiv.), 1,1′-sulfonyldiimidazole (1.5 mmol, 1.5 equiv.), cesium carbonate (1 mmol, 1 equiv.) and THF (4 mL) was added. The reaction mixture was heated at 80 °C for 8 h. After the completion of reaction monitored by TLC, the reaction mixture was filtered through celite and the filtrate was collected and distilled under reduced pressure. The resulting crude product was purified by column chromatography to obtain the 4-methyl-2-oxo-2*H*-chromen-7-yl-1*H*-imidazole-1-sulfonate intermediate **2a** as white solid in 70% yield.

Mp 157–159 °C.

^1^H NMR (400 MHz, CDCl_3_): δ = 2.42 (s, 3H, CH_3_), 6.32 (s, 1H, ArH), 6.87 (dd, *J* = 2.4, 8.8 Hz, 1H, ArH), 6.98 (d, *J* = 2.4 Hz, 1H, ArH), 7.18 (s, 1H, ArH), 7.33 (s, 1H, ArH), 7.59 (d, *J* = 8.8 Hz, 1H, ArH), 7.75 (s, 1H, ArH); ^13^C NMR (100 MHz, CDCl_3_): δ = 18.7 (CH_3_), 110.6 (C-aromatic), 116.0 (C-aromatic), 117.2 (C-aromatic), 118.2 (C-aromatic), 120.2 (C-aromatic), 126.4 (C-aromatic), 131.8 (C-aromatic), 137.5 (C-aromatic), 150.3 (C-aromatic), 151.1 (C-aromatic), 154.1 (C-aromatic), 159.4 (CO); MS (EI): m/z (%) = 306 (24) [M]+, 147 (100); Anal. Calcd for C_13_H_10_N_2_O_5_S: C, 50.98; H, 3.29; N, 9.15; S, 10.47%. Found: C, 50.92; H, 3.29; N, 9.39; S, 10.40%.

### 2.3. Synthesis of Products **4** from Phenols

In a sealed tube with screw cap, (poly)phenols **1a–i** (1 mmol, 1 equiv.), boronic acids **3a–h** (1.1 mmol, 1.1 equiv.), 1,1-sulfonyldiimidazole (1 mmol, 1 equiv.), Na_2_CO_3_ (2 mmol, 2 equiv.) and DMF (2 mL) were added. The reaction mixture was degassed for 10 min under N_2_ atmosphere and then Pd(PPh_3_)_2_Cl_2_ (5 mol%, 0.05 equiv.) was added. The reaction mixture was heated at 90 ℃ for 8 h. After the specified time, the reaction mixture was filtered through celite, the filtrate was diluted with water (10 mL) and extracted thrice with ethyl acetate. The combined organic layers were washed with brine, dried in Na_2_SO_4_ and distilled under reduced pressure to obtain the crude product. The crude product was purified by column chromatography in varying polarities to obtain the titled products **4aa–4ah** and **4bh–4ih** in varying yields. The ^1^H and ^13^C NMR spectra of all the final compounds have been included in the supplementary information.

***7-(4-(1H-pyrazol-1-yl)phenyl)-4-methyl-2H-chromen-2-one (4aa)***

Yield: 82% (248 mg); light yellow solid; mp 160–162 °C.

^1^H NMR (400 MHz, CDCl_3_): δ = 2.48 (d, *J* = 1.2 Hz, 3H, CH_3_), 6.31 (d, *J* = 1.2 Hz, 1H, ArH), 6.51 (t, *J* = 2.4 Hz, 1H, ArH), 7.55–7.57 (m, 2H, ArH), 7.68 (d, *J* = 8.8 Hz, 1H, ArH), 7.72–7.74 (m, 2H, ArH), 7.76 (d, *J* = 1.6 Hz, 1H, ArH), 7.83 (d, *J* = 8.8 Hz, 2H, ArH), 7.99 (d, *J* = 2.8 Hz, 1H, ArH); ^13^C NMR (100 MHz, CDCl_3_): δ = 18.7 (CH_3_), 108.0 (C-aromatic), 115.0 (C-aromatic), 119.1 (C-aromatic), 119.6 (C-aromatic), 122.8 (C-aromatic), 125.1 (C-aromatic), 126.7 (C-aromatic), 128.2 (C-aromatic), 137.0 (C-aromatic), 140.3 (C-aromatic), 141.5 (C-aromatic), 143.7 (C-aromatic), 152.1 (C-aromatic), 154.0 (C-aromatic), 160.8 (CO); MS (EI): *m*/*z* (%) = 302 (100) [M]^+^; Anal. Calcd for C_19_H_14_N_2_O_2_: C, 75.48; H, 4.67; N, 9.27%; Found: C, 75.53; H, 4.42; N, 9.03%.

***4-Methyl-7-(o-tolyl)-2H-chromen-2-one (4ab)***

Yield: 78% (195 mg); white solid; mp 135–137 °C.

^1^H NMR (400 MHz, CDCl_3_): δ = 2.31 (s, 3H, CH_3_), 2.50 (s, 3H, CH_3_), 6.33 (s, 1H, ArH), 7.24–7.29 (m, 3H, ArH), 7.31–7.32 (m, 3H, ArH), 7.67 (d, *J* = 8 Hz, 1H, ArH); ^13^C NMR (100 MHz, CDCl_3_): δ = 18.6 (CH_3_), 20.4 (CH_3_), 114.9 (C-aromatic), 117.6 (C-aromatic), 118.6 (C-aromatic), 124.3 (C-aromatic), 125.5 (C-aromatic), 126.1 (C-aromatic), 128.2 (C-aromatic), 129.5 (C-aromatic), 130.7 (C-aromatic), 135.2 (C-aromatic), 140.0 (C-aromatic), 146.0 (C-aromatic), 152.3 (C-aromatic), 153.4 (C-aromatic), 160.9 (CO); MS (EI): *m*/*z* (%) = 250 (100) [M]^+^; Anal. Calcd for C_17_H_14_O_2_: C, 81.58; H, 5.64%; Found: C, 81.37; H, 5.26%.

***7-(2-Ethoxyphenyl)-4-methyl-2H-chromen-2-one (4ac)***

Yield: 80% (224 mg); white solid; mp 116–118 °C.

^1^H NMR (400 MHz, CDCl_3_): δ = 1.37 (t, *J* = 7.2 Hz, 3H, CH_3_), 2.47 (s, 3H, CH_3_), 4.08 (q, *J* = 7.2 Hz, 2H, CH_2_), 6.29 (s, 1H, ArH), 6.99–7.06 (m, 2H, ArH), 7.33–7.37 (m, 2H, ArH), 7.51 (dd, *J* = 1.6, 8.4 Hz, 1H, ArH), 7.58 (s, 1H, ArH), 7.62 (d, *J* = 8 Hz, 1H, ArH); ^13^C NMR (100 MHz, CDCl_3_): δ = 14.7 (CH_3_), 18.6 (CH_3_), 64.0 (CH_2_), 112.6 (C-aromatic), 114.6 (C-aromatic), 117.8 (C-aromatic), 118.5 (C-aromatic), 121.0 (C-aromatic), 123.9 (C-aromatic), 125.7 (C-aromatic), 128.7 (C-aromatic), 129.7 (C-aromatic), 130.7 (C-aromatic), 142.7 (C-aromatic), 152.4 (C-aromatic), 153.3 (C-aromatic), 155.9 (C-aromatic), 161.2 (CO); MS (EI): *m*/*z* (%) = 280 (100) [M]^+^; Anal. Calcd for C_18_H_16_O_3_: C, 77.12; H, 5.75%. Found: C, 77.33; H, 6.06%.

***7-(3-Fluorophenyl)-4-methyl-2H-chromen-2-one (4ad)***

Yield: 69% (175 mg); white solid; mp 140–143 °C.

^1^H NMR (400 MHz, CDCl_3_): δ = 2.48 (s, 3H, CH_3_), 6.32 (s, 1H, ArH), 7.10–7.14 (m, 1H, ArH), 7.33 (d, *J* = 10 Hz, 1H, ArH), 7.40–7.48 (m, 2H, ArH), 7.51–7.54 (m, 2H, ArH), 7.67 (d, *J* = 8.4 Hz, 1H, ArH); ^13^C NMR (100 MHz, CDCl_3_): δ = 18.7 (CH_3_), 114.2 (d, *J* = 14 Hz, C-aromatic), 115.2 (C-aromatic), 115.3 (C-aromatic), 115.4 (C-aromatic), 119.4 (C-aromatic), 122.9 (d, *J* = 2 Hz, C-aromatic), 123.0 (C-aromatic), 125.1 (C-aromatic), 130.7 (d, *J* = 5 Hz, C-aromatic), 141.4 (d, *J* = 5 Hz, C-aromatic), 143.5 (d, *J* = 1 Hz, C-aromatic), 152.0 (C-aromatic), 154.0 (C-aromatic), 160.7 (CO), 163.2 (d, *J* = 164 Hz, CF); MS (EI): *m*/*z* (%) = 254 (100) [M]^+^; Anal. Calcd for C_16_H_11_FO_2_: C, 75.58; H, 4.36; F, 7.47%. Found: C, 75.80; H, 4.46; F, 7.62%.

***4-Methyl-7-(3-(trifluoromethoxy)phenyl)-2H-chromen-2-one (4ae)***

Yield: 73% (234 mg); white solid; mp 65–67 °C.

^1^H NMR (400 MHz, CDCl_3_): δ = 2.47 (s, 3H, CH_3_), 6.31 (s, 1H, ArH), 7.28 (s, 1H, ArH), 7.46 (s, 1H, ArH), 7.49–7.56 (m, 4H, ArH), 7.68 (d, *J* = 8 Hz, 1H, ArH); ^13^C NMR (100 MHz, CDCl_3_): δ = 18.6 (CH_3_), 115.3 (2 peaks, C-aromatic), 119.5 (C-aromatic), 119.8 (C-aromatic), 120.5 (q, *J* = 250 Hz, CF_3_), 120.7 (C-aromatic), 123.0 (C-aromatic), 125.2 (C-aromatic), 125.5 (C-aromatic), 130.5 (C-aromatic), 141.2 (C-aromatic), 143.1 (C-aromatic), 149.9 (C-aromatic), 151.9 (C-aromatic), 154.0 (C-aromatic), 160.6 (CO); MS (EI): *m*/*z* (%) = 320 (81) [M]^+^, 292 (100); Anal. Calcd for C_17_H_11_F_3_O_3_: C, 63.75; H, 3.46; F, 17.80%. Found: C, 63.35; H, 3.18; F, 17.81%.

***7-(3-Methoxyphenyl)-4-methyl-2H-chromen-2-one (4af)***

Yield: 81% (215 mg); off white solid; mp 131–134 °C.

^1^H NMR (400 MHz, CDCl_3_): δ = 2.46 (s, 3H, CH_3_), 3.88 (s, 3H, OCH_3_), 6.29 (s, 1H, ArH), 6.96 (d, *J* = 8 Hz, 1H, ArH), 7.14 (s, 1H, ArH), 7.21 (d, *J* = 7.2 Hz, 1H, ArH), 7.39 (t, *J* = 8 Hz, 1H, ArH), 7.51–7.53 (m, 2H, ArH), 7.64 (d, *J* = 8.4 Hz, 1H, ArH); ^13^C NMR (100 MHz, CDCl_3_): δ = 18.6 (CH_3_), 55.4 (OCH_3_), 113.0 (C-aromatic), 113.8 (C-aromatic), 114.8 (C-aromatic), 115.2 (C-aromatic), 119.0 (C-aromatic), 119.6 (C-aromatic), 123.1 (C-aromatic), 124.9 (C-aromatic), 130.1 (C-aromatic), 140.6 (C-aromatic), 144.8 (C-aromatic), 152.1 (C-aromatic), 153.9 (C-aromatic), 160.1 (C-aromatic), 160.9 (CO); MS (EI): *m*/*z* (%) = 266 (100) [M]^+^; Anal. Calcd for C_17_H_14_O_3_: C, 76.68; H, 5.30%. Found: C, 77.01; H, 5.12%.

***7-(4-Diethylaminophenyl)-4-methyl-2H-chromen-2-one (4ag)***

Yield: 76% (233 mg); yellow solid; mp 154–157 °C.

^1^H NMR (400 MHz, CDCl_3_): δ = 1.21 (t, *J* = 6.8 Hz, 6H, 2CH_3_), 2.44 (s, 3H, CH_3_), 3.42 (q, *J* = 7.2 Hz, 4H, 2CH_2_), 6.22 (s, 1H, ArH), 6.76 (d, *J* = 8.4 Hz, 2H, ArH), 7.50–7.59 (m, 5H, ArH); ^13^C NMR (100 MHz, CDCl_3_): δ = 12.6 (CH_3_), 18.6 (CH_3_), 44.4 (CH_2_), 111.8 (C-aromatic), 113.3 (C-aromatic), 113.7 (C-aromatic), 117.6 (C-aromatic), 121.8 (C-aromatic), 124.7 (C-aromatic), 125.2 (C-aromatic), 128.1 (C-aromatic), 145.1 (C-aromatic), 148.1 (C-aromatic), 152.4 (C-aromatic), 154.2 (C-aromatic), 161.3 (CO); MS (EI): *m*/*z* (%) = 307 (53) [M]^+^, 292 (100); Anal. Calcd for C_20_H_21_NO_2_: C, 78.15; H, 6.89; N, 4.56%. Found: C, 78.02; H, 6.97; N, 4.37%.

***7-(4-Methoxyphenyl)-4-methyl-2H-chromen-2-one (4ah)***

Yield: 85% (226 mg); off white solid; mp 133–136 °C.

^1^H NMR (400 MHz, CDCl_3_): δ = 2.46 (s, 3H, CH_3_), 3.87 (s, 3H, OCH_3_), 6.27 (s, 1H, ArH), 7.01 (d, *J* = 8.4 Hz, 2H, ArH), 7.49–7.51 (m, 2H, ArH), 7.58 (d, *J* = 8.4 Hz, 2H, ArH), 7.63 (d, *J* = 8.4 Hz, 1H, ArH); ^13^C NMR (100 MHz, CDCl_3_): δ = 18.6 (CH_3_), 55.4 (OCH_3_), 114.4 (C-aromatic), 114.5 (C-aromatic), 114.6 (C-aromatic), 118.4 (C-aromatic), 122.5 (C-aromatic), 124.9 (C-aromatic), 128.3 (C-aromatic), 131.5 (C-aromatic), 144.5 (C-aromatic), 152.2 (C-aromatic), 154.0 (C-aromatic), 160.1 (C-aromatic), 161.0 (CO); MS (EI): *m*/*z* (%) = 266 (100) [M]^+^; Anal. Calcd for C_17_H_14_O_3_: C, 76.68; H, 5.30%. Found: C, 76.71; H, 5.08%.

***7-(4-Methoxyphenyl)-2H-chromen-2-one (4bh)***

Yield: 80% (202mg); off white solid; mp 158–161 °C.

^1^H NMR (400 MHz, CDCl_3_): δ = 3.86 (s, 3H, OCH_3_), 6.39 (d, *J* = 9.6 Hz, 1H, ArH), 7.00 (d, *J* = 8.8 Hz, 2H, ArH), 7.46–7.51 (m, 3H, ArH), 7.56 (d, *J* = 8.8 Hz, 2H, ArH), 7.71 (d, *J* = 9.6 Hz, 1H, ArH); ^13^C NMR (100 MHz, CDCl_3_): δ = 55.4 (OCH_3_), 114.4 (C-aromatic), 114.6 (C-aromatic), 115.9 (C-aromatic), 117.3 (C-aromatic), 122.8 (C-aromatic), 128.1 (C-aromatic), 128.4 (C-aromatic), 131.5 (C-aromatic), 143.2 (C-aromatic), 144.7 (C-aromatic), 154.6 (C-aromatic), 160.2 (C-aromatic), 161.0 (CO); MS (EI): *m*/*z* (%) = 252 (100) [M]^+^; Anal. Calcd for C_16_H_12_O_3_: C, 76.18; H, 4.79%. Found: C, 76.42; H, 4.65%.

***7-(4-Methoxyphenyl)-4-(trifluoromethyl)-2H-chromen-2-one (4ch)***

Yield: 75% (240 mg); off white solid; mp 154–157 °C.

^1^H NMR (400 MHz, CDCl_3_): δ = 3.88 (s, 3H, OCH_3_), 6.77 (s, 1H, ArH), 7.03 (d, *J* = 8.4 Hz, 2H, ArH), 7.56–7.61 (m, 4H, ArH), 7.76 (d, *J* = 8 Hz, 1H, ArH); ^13^C NMR (100 MHz, CDCl_3_): δ = 55.4 (OCH_3_), 111.9 (C-aromatic), 114.7 (C-aromatic), 114.8 (C-aromatic), 115.0 (q, *J* = 6 Hz, C-aromatic), 121.6 (q, *J* = 274 Hz, CF_3_), 123.3 (C-aromatic), 125.6 (C-aromatic), 128.4 (C-aromatic), 130.7 (C-aromatic), 141.4 (q, *J* = 33 Hz, C-aromatic), 145.8 (C-aromatic), 154.9 (C-aromatic), 159.1 (C-aromatic), 160.6 (CO); MS (EI): *m*/*z* (%) = 320 (100) [M]^+^; Anal. Calcd for C_17_H_11_F_3_O_3_: C, 63.75; H, 3.46; F, 17.80%. Found: C, 63.70; H, 3.81; F, 17.93%.

***Ethyl-2-(7-(4-methoxyphenyl)-2-oxo-2H-chromen-4-yl)acetate (4dh)***

Yield: 74% (250 mg); off white solid; mp 130–133 °C.

^1^H NMR (400 MHz, CDCl_3_): δ = 1.27 (t, *J* = 7.2 Hz, 3H, CH_3_), 3.78 (s, 2H, CH_2_), 3.86 (s, 3H, CH_3_), 4.21 (q, *J* = 7.2 Hz, 2H, OCH_2_), 6.37 (s, 1H, ArH), 7.01 (d, *J* = 8.4 Hz, 2H, ArH), 7.49–7.51 (m, 2H, ArH), 7.56–7.62 (m, 3H, ArH); ^13^C NMR (100 MHz, CDCl_3_): δ = 14.1 (CH_3_), 38.2 (CH_2_), 55.4 (OCH_3_), 61.8 (OCH_2_), 114.6 (C-aromatic), 114.7 (C-aromatic), 116.3 (C-aromatic), 117.3 (C-aromatic), 122.7 (C-aromatic), 124.9 (C-aromatic), 128.3 (C-aromatic), 131.3 (C-aromatic), 144.8 (C-aromatic), 147.9 (C-aromatic), 154.3 (C-aromatic), 160.2 (C-aromatic), 160.6 (CO), 168.7 (CO); MS (EI): *m*/*z* (%) = 338 (100) [M]^+^; Anal. Calcd for C_20_H_18_O_5_: C, 70.99; H, 5.36%. Found: C, 71.30; H, 5.22%.

***9-(4-Methoxyphenyl)-1-methyl-3H-benzo[f]chromen-3-one (4eh)***

Yield: 78% (246 mg); light yellow solid; mp 174–177 °C.

^1^H NMR (400 MHz, CDCl_3_): δ = 3.01 (s, 3H, CH_3_), 3.89 (s, 3H, OCH_3_), 6.40 (s, 1H, ArH), 7.06 (d, *J* = 8.4 Hz, 2H, ArH), 7.46 (d, *J* = 8.8 Hz, 1H, ArH), 7.64 (d, *J* = 8.4 Hz, 2H, ArH), 7.77 (d, *J* = 8.4 Hz, 1H, ArH), 7.97 (t, *J* = 8.8 Hz, 3H, ArH), 8.75 (s, 1H, ArH); ^13^C NMR (100 MHz, CDCl_3_): δ = 26.5 (CH_3_), 55.4 (OCH_3_), 114.6 (C-aromatic), 116.5 (C-aromatic), 117.5 (C-aromatic), 122.8 (C-aromatic), 124.8 (C-aromatic), 128.6 (C-aromatic), 130.1 (2 peaks, C-aromatic), 130.7 (C-aromatic), 133.3 (C-aromatic), 133.5 (C-aromatic), 140.4 (C-aromatic), 154.0 (C-aromatic), 155.1 (C-aromatic), 159.7 (C-aromatic), 160.4 (CO); MS (EI): *m*/*z* (%) = 316 (100) [M]^+^; Anal. Calcd for C_21_H_16_O_3_: C, 79.73; H, 5.10%. Found: C, 79.98; H, 5.34%.

***6-Acetyl-7-(4-methoxyphenyl)-4-methyl-2H-chromen-2-one (4fh)***

Yield: 75% (231 mg); off white solid; mp 180–182 °C.

^1^H NMR (600 MHz, CDCl_3_): δ = 2.00 (s, 3H, CH_3_), 2.48 (s, 3H, CH_3_), 3.87 (s, 3H, OCH_3_), 6.33 (s, 1H, ArH), 7.00 (d, *J* = 7.8 Hz, 2H, ArH), 7.29 (d, *J* = 7.8 Hz, 2H, ArH), 7.32 (s, 1H, ArH), 7.79 (s, 1H, ArH); ^13^C NMR (150 MHz, CDCl_3_): δ = 18.7 (CH_3_), 30.4 (CH_3_), 55.4 (OCH_3_), 114.6 (C-aromatic), 115.5 (C-aromatic), 118.1 (C-aromatic), 118.6 (C-aromatic), 125.2 (C-aromatic), 130.0 (C-aromatic), 131.3 (C-aromatic), 137.2 (C-aromatic), 144.1 (C-aromatic), 152.1 (C-aromatic), 154.5 (C-aromatic), 160.2 (C-aromatic), 160.4 (CO), 203.6 (CO); MS (EI): *m*/*z* (%) = 308 (100) [M]^+^; Anal. Calcd for C_19_H_16_O_4_: C, 74.01; H, 5.23%. Found: C, 73.61; H, 5.11%.

***7-Methoxy-3-(4′-methoxy [1,1′-biphenyl]-4-yl)chroman (4gh)***

Yield: 68% (235 mg); white solid; mp 157–160 °C.

^1^H NMR (400 MHz, CDCl_3_): δ = 3.02 (d, *J* = 9.6 Hz, 2H, CH_2_), 3.26–3.28 (m, 1H, CH), 3.79 (s, 3H, OCH_3_), 3.86 (s, 3H, OCH_3_), 4.06 (t, *J* = 10.4 Hz, 1H, CH), 4.38 (d, *J* = 10.4 Hz, 1H, CH), 6.46 (s, 1H, ArH), 6.50 (d, *J* = 8.4 Hz, 1H, ArH), 6.98–7.02 (m, 3H, ArH), 7.30 (d, *J* = 8 Hz, 2H, ArH), 7.52–7.55 (m, 4H, ArH); ^13^C NMR (100 MHz, CDCl_3_): δ = 31.7 (CH_2_), 38.4 (CH), 55.4 (2 peaks, OCH_3_), 70.9 (CH_2_), 101.4 (C-aromatic), 107.4 (C-aromatic), 114.1 (C-aromatic), 114.3 (C-aromatic), 127.1 (C-aromatic), 127.8 (C-aromatic), 128.1 (C-aromatic), 130.2 (C-aromatic), 133.3 (C-aromatic), 139.7 (C-aromatic), 139.8 (C-aromatic), 155.1 (C-aromatic), 159.2 (2 peaks, C-aromatic); MS (EI): *m*/*z* (%) = 346 (45) [M]^+^, 210 (100); Anal. Calcd for C_23_H_22_O_3_: C, 79.74; H, 6.40%. Found: C, 79.48; H, 6.49%.

***3,7-Bis(4-methoxyphenyl)chromane (4hh)***

Yield: 70% (242 mg); white solid; mp 160–162 °C.

^1^H NMR (400 MHz, CDCl_3_): δ = 3.04 (d, *J* = 8 Hz, 2H, CH_2_), 3.23–3.28 (m, 1H, CH), 3.81 (s, 3H, OCH_3_), 3.85 (s, 3H, OCH_3_), 4.03 (t, *J* = 10.8 Hz, 1H, CH), 4.36 (d, *J* = 10.8 Hz, 1H, CH), 6.91 (d, *J* = 8.4 Hz, 2H, ArH), 6.97 (d, *J* = 8.4 Hz, 2H, ArH), 7.07–7.15 (m, 3H, ArH), 7.19 (d, *J* = 8.8 Hz, 2H, ArH), 7.52 (d, *J* = 8.8 Hz, 2H, ArH); ^13^C NMR (100 MHz, CDCl_3_): δ = 32.4 (CH_2_), 37.9 (CH), 55.3 (2 peaks, OCH_3_), 71.2 (CH_2_), 114.3 (C-aromatic), 114.4 (C-aromatic), 114.6 (C-aromatic), 118.9 (C-aromatic), 120.5 (C-aromatic), 128.0 (C-aromatic), 128.3 (C-aromatic), 130.0 (C-aromatic), 133.5 (2 peaks, C-aromatic), 140.4 (C-aromatic), 154.7 (C-aromatic), 158.8 (C-aromatic), 159.3 (C-aromatic); MS (EI): *m*/*z* (%) = 346 (63) [M]^+^, 134 (100); Anal. Calcd for C_23_H_22_O_3_: C, 79.74; H, 6.40%. Found: C, 80.13; H, 6.51%.

***2-(4-Methoxyphenyl)naphthalene (4ih)***

Yield: 85% (199 mg); off white solid; mp 140–143 °C.

^1^H NMR (400 MHz, CDCl_3_): δ = 3.88 (s, 3H, OCH_3_), 7.03 (d, *J* = 8.8 Hz, 2H, ArH), 7.45–7.52 (m, 2H, ArH), 7.67 (d, *J* = 8.4 Hz, 2H, ArH), 7.73 (d, *J* = 8.4 Hz, 1H, ArH), 7.85–7.91 (m, 3H, ArH), 8.00 (s, 1H, ArH); ^13^C NMR (100 MHz, CDCl_3_): δ = 55.4 (OCH_3_), 114.3 (C-aromatic), 125.1 (C-aromatic), 125.5 (C-aromatic), 125.7 (C-aromatic), 126.2 (C-aromatic), 127.6 (C-aromatic), 128.1 (C-aromatic), 128.4 (2 peaks, C-aromatic), 132.3 (C-aromatic), 133.7 (C-aromatic), 133.8 (C-aromatic), 138.2 (C-aromatic), 159.3 (C-aromatic); MS (EI): *m*/*z* (%) = 234 (100) [M]^+^; Anal. Calcd for C_17_H_14_O: C, 87.15; H, 6.02%. Found: C, 87.17; H, 6.05%.

### 2.4. Procedure for Determining Antioxidant Potential of the Synthesized Compounds

The conventional colorimetric DPPH• scavenging capacity assay was carried out according to a previously reported laboratory protocol [29]. Briefly, a 100 µL (100 µg concentration) sample of organic compounds prepared in methanol was added to 3 mL of 0.004% w/v DPPH• solution. Each test tube was made up to a 4 mL final volume. The reference standard BHT was also dissolved in methanol to make the same concentration as that of the tested compounds. Each mixture was vortexed for some time and left to stand in the dark for 10 min at ambient temperature. The absorbance of each reaction mixture was measured at 517 nm against a blank of methanol using a UV-visible spectrometer (Shimadzu UV-1800). The level of DPPH• remaining for each experiment was calculated by the following equation:

$$\%\mathrm{Scavenging}\mathrm{Activity}=\frac{\mathrm{Absorbance}\mathrm{of}\mathrm{the}\mathrm{control}-\mathrm{Absorbance}\mathrm{of}\mathrm{the}\mathrm{test}\mathrm{sample}}{\mathrm{Absorbance}\mathrm{of}\mathrm{the}\mathrm{control}}\times 100$$

The inhibition curve was plotted for triplicate experiments and represented as percentage of mean inhibition ± standard deviation.

## 3. Results

### 3.1. Chemistry and Pharmacological Studies

#### 3.1.1. Synthesis of Coumarin Derivatives by One-Pot Suzuki Coupling

**Scheme 1:**
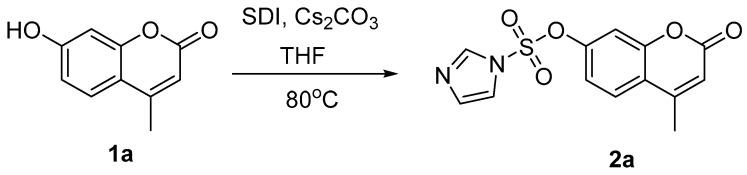
Synthesis of coumarin imidazylate intermediate.

As illustrated in Scheme 1, our synthetic strategy started from preparing the coumarin imidazylate intermediate **2a** from 4-methyl-7-hydroxy coumarin **1a** in the presence of 1,1-sulfonyldiimidazole (SDI) and cesium carbonate in THF solvent at 80 °C. This intermediate was then planned to react with different arylboronic acids for synthesizing a series of 4-methyl-7-substituted coumarins by Suzuki coupling.

As a model reaction, we took **2a** and (4-(1*H*-pyrazol-1-yl)phenyl)boronic acid **3a** to optimize the reaction conditions. A series of palladium catalysts, ligands, base and solvents were screened in our optimization studies (Table 1). Gratifyingly, we obtained the expected product **4aa** in 85% isolated yield when the reaction was carried out at 90 °C in DMF solvent by employing PdCl_2_(PPh_3_)_2_ as the catalyst and Na_2_CO_3_ as base. All other catalyst-ligand combinations rendered the desired product in lower yields. Among the various bases screened, cesium carbonate procured the required product in slightly better yield. However, Na_2_CO_3_ was found to be better than Cs_2_CO_3_ and other organic bases for this reaction. Similarly, DMF was found to be the best solvent for our reactions when compared to dioxane, water and THF. The reaction was found to be sluggish at 60 °C and a slightly lower yield of the expected product was obtained at 110 °C.

After the detailed optimization studies, our next task was to evaluate the substrate scope by synthesizing an array of 4-methyl-7-substituted coumarin derivatives. However, we planned to do some control experiments to explore the possibility of developing a one-pot protocol for converting 4-methyl-7-hydroxy coumarin **1a** to Suzuki coupled product **4aa**. The feasibility of our optimized Suzuki coupling reaction in the Cs_2_CO_3_ base and THF solvent (albeit in slightly lower yield) further encouraged us to screen some conditions for one-pot methodology. Accordingly, we treated **1a** with boronic acid **3a**, SDI and PdCl_2_(PPh_3_)_2_ catalyst in different bases and solvents (Table 2). To our delight, we obtained the desired product **4aa** in 82% isolated yield when the reaction was carried out in DMF at 90 °C. Even though the yield of the isolated product was slightly lower than the previously optimized two-step methodology, this one-pot protocol was found to be facile, convenient and step-economic.

**Scheme 2:**
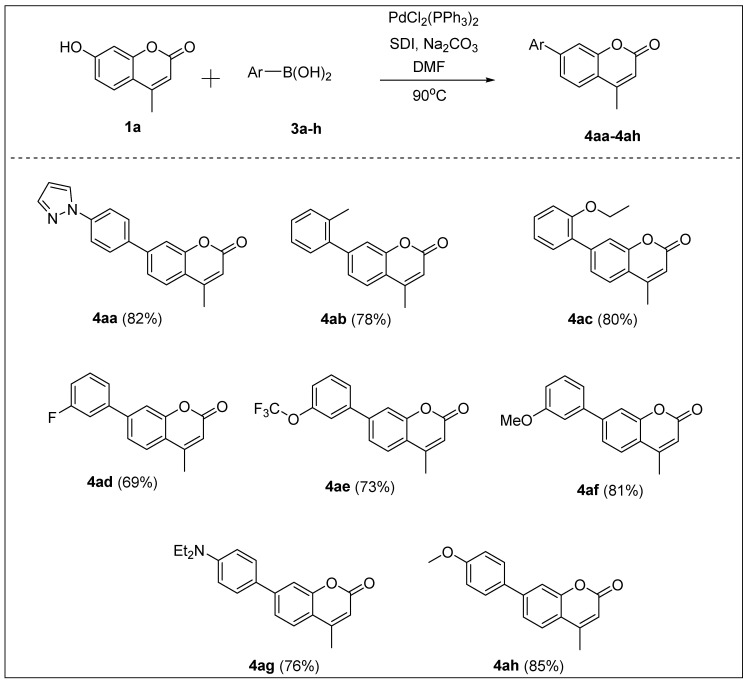
Scope of boronic acids in one-pot synthesis.

After the successful development of one-pot synthesis, we shifted our attention to evaluate the substrate scope. Accordingly, 4-methyl-7-hydroxy coumarin **1a** was treated with diverse arylboronic acids **3a–h** in view of synthesizing an assortment of 4-methyl-7-substituted coumarin derivatives (Scheme 2). Gratifyingly, all the boronic acids reacted well enough to procure the expected products **4aa–4ah** in good to acceptable yields (69–85% isolated yield). Later, it was planned to evaluate the antioxidant potential of these synthesized compounds by DPPH assay.

#### 3.1.2. Antioxidant Activity of Coumarin Derivatives **4aa–4ah**

One of the most effective methods for evaluating the antioxidant potential of organic compounds is DPPH assay. The radical scavenging activity can be easily determined in terms of percentage inhibition by this assay [30]. Accordingly, we evaluated the antioxidant capacity of the synthesized coumarin derivatives **4aa–4ah** by DPPH radical scavenging activity studies [31]. Butylated hydroxytoluene (BHT) was used as the reference standard in our investigation. It is worth noting that the coumarin derivatives have an extended п-conjugated system that could possibly be favorable for enhanced antioxidant potential. The percentage inhibition at 100 µg concentration has been evaluated and our results are summarized in Table 3.

From our studies, the reference standard BHT exhibited a strong antioxidant activity of 90.4% at 100 µg concentration. Among the compounds screened, **4ah** showed the highest radical scavenging capacity of 81.7% at 100 µg concentration. However, the compounds **4aa** (75.3%), **4af** (77.6%) and **4ag** (76%) also demonstrated promising antioxidant potential at the same concentration. To our disappointment, the compounds **4ad** and **4ae** exhibited significantly lower potency. Other tested compounds in this series, such as **4ab** and **4ac,** possessed moderate radical scavenging activity, which indicates the possibility of their improved potential at higher concentrations.

#### 3.1.3. Synthesis of Coumarin- and Equol-Based Compounds

**Scheme 3:**
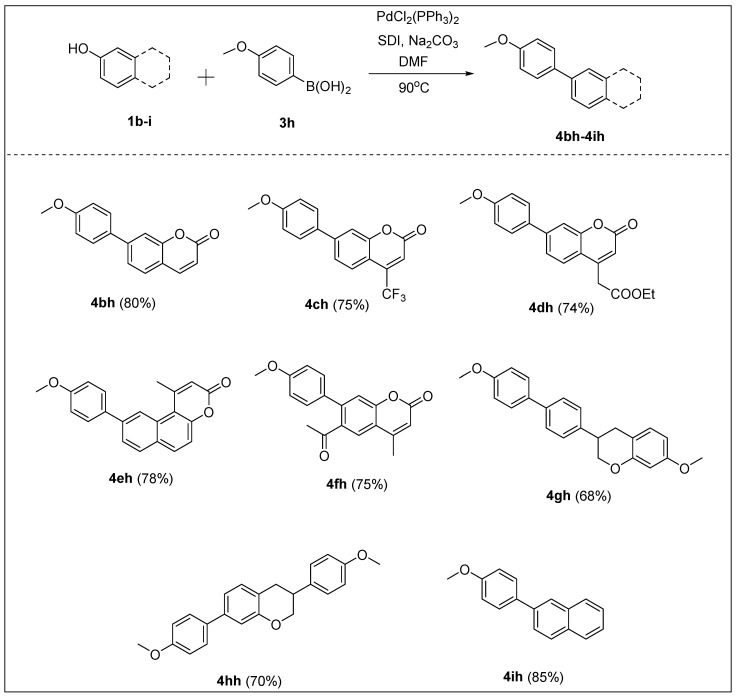
Scope of (poly)phenols in one-pot synthesis.

After identifying **4ah** as the most potent antioxidant among the tested compounds, it was planned to synthesize some additional compounds from available (poly)phenols. Accordingly, different (poly)phenols based on coumarins and equols **1b–i** were treated with 4-methoxyphenylboronic acid **3h** in our optimized one-pot Suzuki coupling conditions (Scheme 3). Fortunately, we obtained the required final products **4bh–4ih** in good to satisfactory yields (68–85% isolated yield). The antioxidant potential of these synthesized compounds was then evaluated by DPPH assay.

#### 3.1.4. Antioxidant Activity of **4bh–4ih** by DPPH Assay

After the successful synthesis of the coumarin- and equol-based final products, **4bh–4ih**, the antioxidant capacity of these molecules was subsequently determined by DPPH assay employing BHT as the standard (Table 4). From our results, we identified the need for the presence of electron-donating functionalities in enhancing the antioxidant activity. Among the compounds screened, **4eh**, **4gh** and **4hh** showed promising potential that was comparable to the reference standard, BHT. The compounds **4ch**, **4dh** and **4fh** exhibited lower potency when compared with other tested compounds. Moreover, the compounds **4bh** and **4ih** displayed moderate antioxidant potential. The SAR studies were then carried out to understand the relationship between the antioxidant capacity and the structural features of the tested compounds.

## 4. Discussion

### 4.1. Antioxidant Activity of Phenolic Compounds **1a–i** by DPPH Assay

In order to understand the actual need for derivatization of phenolic compounds by Suzuki coupling for developing novel antioxidants, we decided to determine the antioxidant activity evaluation of the parent phenolic compounds **1a–i** by DPPH assay. The results of antioxidant screening of **1a–i** has been illustrated in Table 5. The phenolic compound **1g** displayed the highest antioxidant potential (80.3%); however, the corresponding Suzuki coupled product **4gh** showed better antioxidant activity (83.8%). It is noteworthy that the parent phenol **1b** possessed better antioxidant potential when compared to the corresponding Suzuki coupled product **4bh**. Nevertheless, most of the phenolic compounds **1a–i** displayed lower antioxidant potential when compared to the Suzuki coupled products **4ah–4ih,** which highlights the need for derivatization of parent phenolic compounds.

### 4.2. SAR Studies

**Figure 1:**
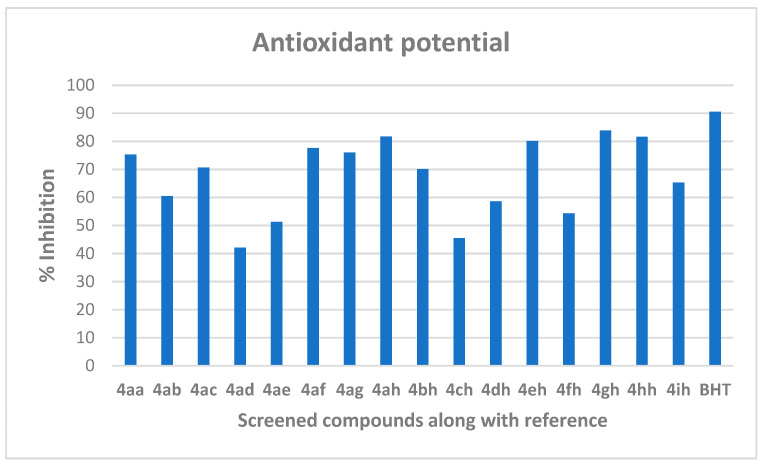
Results of antioxidant screening of synthesized compounds.

The structure activity determination (SAR) studies help us to understand the importance of some critical structural features in enhancing the overall pharmacological potential of the tested compounds. In this communication, we have reported the step-economic one-pot synthesis of coumarin- and equol-based compounds and the evaluation of their biological potential as antioxidants. The results of free radical scavenging capacity of all the compounds tested have been summarized in Figure 1. Some of the compounds such as **4ah**, **4eh**, **4gh** and **4hh** exhibited comparable antioxidant capacity. The SAR studies were carried out to get more insights about the profound antioxidant activity of these promising compounds.

From the SAR studies, it was found that the compounds **4ah**, **4gh** and **4hh** contain the electron-donating methoxy group, and the compound **4eh** has three aromatic rings fused together. Moreover, the most promising compounds **4gh** and **4hh** comprises two electron-donating methoxy groups. Hence, in this study, the presence of electron-donating functionalities and extended п-conjugation are crucial for enhancing the radical scavenging activity of the tested compounds. Moreover, the compounds **4ad**, **4ae 4ch**, **4dh** and **4fh,** containing electron-withdrawing groups, displayed lower activity profiles. It is presumed that the hydrophilic electron-donating functionalities enable the stabilization of the oxygen-centered radical and thereby reduce the O–H bond dissociation enthalpy (BDE). This will possibly increase the radical scavenging activity by abstraction of hydrogen [32,33]: a plausible reason for the promising antioxidant potential of **4ah**, **4gh** and **4hh** when compared to other synthesized molecules.

## 5. Conclusions

In summary, we have developed a facile, convenient and step-economic one-pot protocol for direct conversion of (poly)phenols based on coumarin and equol to various (hetero)aryl compounds by palladium-catalyzed Suzuki coupling reaction. The antioxidant capacity of the synthesized compounds was evaluated by DPPH assay, employing BHT as the reference. Among the compounds screened, **4ah**, **4eh**, **4gh** and **4hh** were found to be the most potent ones as they exhibited comparable activity with the employed reference standard. The parent phenolic compounds were also subjected to antioxidant screening for understanding the actual need for their derivatization by Suzuki coupling. Most of the phenolic compounds displayed a slightly lower activity profile when compared to the newly synthesized Suzuki coupled derivatives. The more active compounds were subjected to SAR studies, which revealed the significance of the presence of electron-donating substituents in increasing their overall antioxidant properties. The synthesis of additional compounds, including various natural product derivatives from oils and fats, focusing on the development of new antioxidants with improved potency is currently in process in our laboratory.

## Figures and Tables

**Table 1:** Optimization of reaction conditions for Suzuki coupling ^1^.

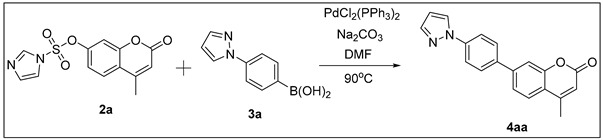					
Entry	Catalyst	Ligand	Base	Solvent	Yield ^2^ 4aa (%)
1	PdCl_2_^.^(PPh_3_)_2_	----	Na_2_CO_3_	DMF	85
2	Pd(OAc)_2_	----	Na_2_CO_3_	DMF	trace
3	Pd(OAc)_2_	Xantphos	Na_2_CO_3_	DMF	55
4	Pd(dppf)Cl_2_	----	Na_2_CO_3_	DMF	60
5	Pd(OAc)_2_	BINAP	Na_2_CO_3_	DMF	40
6	PdCl_2_^.^(PPh_3_)_2_	----	Cs_2_CO_3_	DMF	70
7	PdCl_2_^.^(PPh_3_)_2_	----	K_3_PO_4_	DMF	40
8	PdCl_2_^.^(PPh_3_)_2_	----	Et_3_N	DMF	55
9	PdCl_2_^.^(PPh_3_)_2_	----	DBU	DMF	60
10	PdCl_2_^.^(PPh_3_)_2_	----	Na_2_CO_3_	THF	68
11	PdCl_2_^.^(PPh_3_)_2_	----	Na_2_CO_3_	1,4-Dioxane	25
12	PdCl_2_^.^(PPh_3_)_2_	----	Na_2_CO_3_	H_2_O	40
13	PdCl_2_^.^(PPh_3_)_2_	----	Na_2_CO_3_	1,4-Dioxane-H_2_O (1:1)	60
14 ^3^	PdCl_2_^.^(PPh_3_)_2_	----	Na_2_CO_3_	DMF	50
15 ^4^	PdCl_2_^.^(PPh_3_)_2_	----	Na_2_CO_3_	DMF	80

^1^ Reaction conditions: **2a** (1 mmol), **3a** (1.1 mmol), catalyst (5 mol%), ligand (10 mol%), base (2 mmol), 2 mL solvent at given temperature for 8 h. ^2^ Isolated yield. ^3^ Reaction at 60 °C. ^4^ Reaction at 110 °C.

**Table 2:** Optimization of reaction conditions for one-pot Suzuki coupling ^1^.

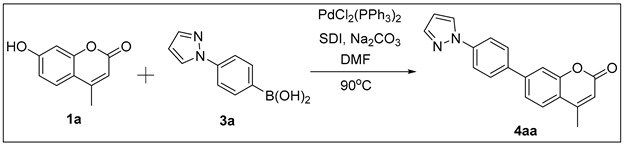		
Entry	Deviation from Standard Conditions	Yield ^2^ 4aa (%)
1	None	82
2	Cs_2_CO_3_ instead of Na_2_CO_3_	78
3	Et_3_N instead of Na_2_CO_3_	60
4	THF instead of DMF	70
5	Reaction at 80 °C	70
6	Reaction at 100 °C	75

^1^ Reaction conditions: **2a** (1 mmol), **3a** (1.1 mmol), SDI (1 mmol), PdCl_2_(PPh_3_)_2_ (5 mol%), base (2 mmol), 2 mL solvent at given temperature for 8 h. ^2^ Isolated yield.

**Table 3:** Determination of the antioxidant activity of the synthesized compounds.

Entry	Compound	% Inhibition at 100 µg Concentration
1	**4aa**	75.3
2	**4ab**	60.5
3	**4ac**	70.6
4	**4ad**	42.1
5	**4ae**	51.3
6	**4af**	77.6
7	**4ag**	76.0
8	**4ah**	81.7
9	Standard (BHT)	90.4

**Table 4:** Determination of antioxidant activity of the synthesized compounds **4bh–4ih**.

Entry	Compound	% Inhibition at 100 µg Concentration
1	**4bh**	70.1
2	**4ch**	45.5
3	**4dh**	58.6
4	**4eh**	80.1
5	**4fh**	54.3
6	**4gh**	83.8
7	**4hh**	81.6
8	**4ih**	65.3
9	Standard (BHT)	90.6

**Table 5:** Determination of antioxidant activity of phenolic compounds **1a–i**.

Entry	Compound	% Inhibition at 100 µg Concentration
1	**1a**	79.4
2	**1b**	73.8
3	**1c**	40.3
4	**1d**	54.8
5	**1e**	77.3
6	**1f**	51.0
7	**1g**	80.3
8	**1h**	79.6
9	**1i**	60.0
10	Standard (BHT)	90.4

## Data Availability

Data are contained within the article and Supplementary Materials.

## Supplementary Materials

The following supporting information can be downloaded at: https://www.mdpi.com/article/10.3390/antiox13101198/s1, File S1: The ^1^H and ^13^C spectra of intermediate **2a** and all final compounds.

## References

[1] Sugamura K., Keaney J.F. (2011). Reactive oxygen species in cardiovascular disease. Free Radical Biol. Med..

[2] Yang Y., Liu Q.W., Shi Y., Song Z.G., Jin Y.H., Liu Z.Q. (2014). Design and synthesis of coumarin-3-acylamino derivatives to scavenge radicals and to protect DNA. Eur. J. Med. Chem..

[3] Mena S., Ortega A., Estrela J.M. (2009). Oxidative stress in environmental-induced carcinogenesis. Mutat. Res..

[4] Fraga C.G., Oteiza P.I. (2011). Dietary flavonoids: Role of (−)-epicatechin and related procyanidins in cell signaling. Free Radical Biol. Med..

[5] Lonn M.E., Dennis J.M., Stocker R. (2012). Actions of “antioxidants” in the protection against atherosclerosis. Free Radical Biol. Med..

[6] Riganti C., Gazzano E., Polimeni M., Aldieri E., Ghigo D. (2012). The pentose phosphate pathway: An antioxidant defense and a crossroad in tumor cell fate. Free Radical Biol. Med..

[7] Rahman I. (2012). Pharmacological antioxidant strategies as therapeutic interventions for COPD. Biochim. Biophys. Acta..

[8] El-Saadony M.T., Yang T., Saad A.M., Alkafaas S.S., Elkafas S.S., Eldeeb G.S., Mohammed D.M., Salem H.M., Korma S.A., Loutfy S.A. (2024). Polyphenols: Chemistry, bioavailability, bioactivity, nutritional aspects and human health benefits: A review. Int. J. Biol. Macromol..

[9] Fatima A., Khan M.S., Ahmad M.W. (2020). Therapeutic potential of equol: A comprehensive review. Curr. Pharm. Des..

[10] Fernandez-Pena L., Matos M.J., Lopez E. (2023). Recent advances in biologically active coumarins from marine sources: Synthesis and evaluation. Mar. Drugs..

[11] Zou Y., Teng Y., Li J., Yan Y. (2024). Recent advances in the biosynthesis of coumarin and its derivatives. Green. Chem. Eng..

[12] Sharma M., Vyas V.K., Bhatt S., Ghate M.D. (2022). Therapeutic potential of 4-substituted coumarins: A conspectus. Eur. J. Med. Chem..

[13] Ghosh S., Ghosh A., Rajanan A., Suresh A.J., Raut P.S., Kundu S., Sahu B.D. (2022). Natural coumarins: Preclinical evidence-based potential candidates to alleviate diabetic nephropathy. Phytomed. Plus..

[14] Nasab N.H., Azimian F., Kruger H.G., Kim S.J. (2022). Acetylcoumarin in cyclic and heterocyclic-containing coumarins: Synthesis and biological applications. Tetrahedron.

[15] Hinman J.W., Hoeksema H., Caron E.L., Jackson W.G. (1956). The partial structure of novobiocin (streptonivicin). J. Am. Chem. Soc..

[16] Kawaguchi H., Tsukiura H., Okanishi M., Miyaki T., Ohmori T., Fujisawa K., Koshiyama H. (1965). Studies on coumermycin, a new antibiotic. Production, isolation and characterization of coumermycin A1. J. Antibiot. Ser. A.

[17] Salvador J., Tassies T., Reverter L., Marco M. (2018). Enzyme-linked immunosorbent assays for therapeutic drug monitoring coumarin oral anticoagulants in plasma. Anal. Chim. Acta..

[18] Dabhi R.C., Sharma V.S., Arya P.S., Patel U.P., Shrivastav P.S., Maru J.J. (2023). Coumarin functionalized dimeric mesogens for promising anticoagulant activity: Tuning of liquid crystalline property. J. Mol. Struct..

[19] Setchell K.D., Brown N.M., Lydeking-Olsen E. (2002). The clinical importance of the metabolite equol-a clue to the effectiveness of soy and its isoflavones. J. Nutr..

[20] Atkinson C., Frankenfeld C.L., Lampe J.W. (2005). Gut bacterial metabolism of the soy isoflavone daidzein: Exploring the relevance to human health. Exp. Biol. Med..

[21] Innocenti M.D., Schreiner T., Breinbauer R. (2023). Recent advances in Pd-catalyzed Suzuki-Miyaura cross-coupling reactions with triflates or nonaflates. Adv. Synth. Catal..

[22] Farhang M., Akbarzadeh A.R., Rabbani M., Ghadiri A.M. (2022). A retrospective-prospective review of Suzuki–Miyaura reaction: From cross-coupling reaction to pharmaceutical industry applications. Polyhedron.

[23] Baviskar B.A., Ajmire P.V., Chumbhale D.S., Khan M.S., Kuchake V.G., Singupuram M., Laddha P.R. (2023). Recent advances in nickel catalyzed Suzuki-Miyaura cross coupling reaction via C-O & C-N bond activation. Sustain. Chem. Pharm..

[24] Joy M.N., Sajith A.M., Santra S., Bhattacherjee D., Beliaev N., Zyryanov G.V., Eltsov O.S., Haridas K.R., Alshammari M.B. (2024). Suzuki–Miyaura coupling of aryl fluorosulfates in water: A modified approach for the synthesis of novel coumarin derivatives under mild conditions. J. Taibah Univ. Sci..

[25] Joy M.N., Bodke Y.D., Telkar S., Bakulev V.A. (2020). Synthesis of coumarins linked with 1,2,3-triazoles under microwave irradiation and evaluation of their antimicrobial and antioxidant activity. J. Mex. Chem. Soc..

[26] Joy M.N., Guda M.R., Zyryanov G.V. (2023). Evaluation of anti-inflammatory and anti-tubercular activity of 4-methyl-7-substituted coumarin hybrids and their structure activity relationships. Pharmaceuticals.

[27] Rishikesan R., Karuvalam R.P., Muthipeedika N.J., Sajith A.M., Eeda K.R., Pakkath R., Haridas K.R., Bhaskar V., Narasimhamurthy K.H., Muralidharan A. (2021). Synthesis of some novel piperidine fused 5-thioxo-1*H*-1,2,4-triazoles as potential antimicrobial and antitubercular agents. J. Chem. Sci..

[28] Fulmer G.R., Miller A.J.M., Sherden N.H., Gottlieb H.E., Nudelman A., Stoltz B.M., Bercaw J.E., Goldberg K.I. (2010). NMR chemical shifts of trace impurities: Common laboratory solvents, organics, and gases in deuterated solvents relevant to the organometallic chemist. Organometallics..

[29] Braca A., Tommasi N.D., Bari L.D., Pizza C., Politi M., Morelli I. (2001). Antioxidant principles from bauhinia tarapotensis. J. Nat. Prod..

[30] Niki E. (1987). Antioxidants in relation to lipid peroxidation. Chem. Phys. Lipids.

[31] Matos M.J., Pérez-Cruz F., Vazquez-Rodriguez S., Uriarte E., Santana L., Borges F., Olea-Azar C. (2013). Remarkable antioxidant properties of a series of hydroxy-3-arylcoumarins. Bioorg Med. Chem..

[32] Yamagami C., Akamatsu M., Motohashi N., Hamada S., Tanahashi T. (2005). Quantitative structure–activity relationship studies for antioxidant hydroxybenzalacetones by quantum chemical and 3-D-QSAR(CoMFA) analyses. Bioorg Med. Chem. Lett..

[33] Sivakumar P.M., Prabhakar P.K., Doble M. (2011). Synthesis, antioxidant evaluation, and quantitative structure–activity relationship studies of chalcones. Med. Chem. Res..

