# Supplementary material for: Facile One-Pot Conversion of (poly)phenols to Diverse (hetero)aryl Compounds by Suzuki Coupling Reaction: A Modified Approach for the Synthesis of Coumarin- and Equol-Based Compounds as Potential Antioxidants
Source: Antioxidants (Basel). 2024 Oct 3;13(10):1198. doi: 10.3390/antiox13101198 (PMC11504026; doi:10.3390/antiox13101198)

Supporting Information

Facile one pot conversion of (poly)phenols to diverse (hetero)aryl compounds by Suzuki coupling reaction: A modified approach for the synthesis of coumarin and equol based compounds as potential antioxidants

Muthipeedika Nibin Joy [1*], Igor S. Kovalev [1], Olga V. Shabunina [1], Sougata Santra [1] and Grigory V. Zyryanov [1, 2]

[1] Ural Federal University, 19 Mira Street, Yekaterinburg, 620002, Russia.

[2] I. Ya. Postovskiy Institute of Organic Synthesis, Ural Division of the Russian Academy of Sciences, 22 S. Kovalevskoy Street, Yekaterinburg, 620219, Russia.

*Corresponding author: mnibinjoy@gmail.com

SPECTRA

^{1}H NMR and ^{13}C NMR spectra of the synthesized products: NMR spectra were recorded in $CDCl_3$ or DMSO-*d*$_6$.

^{1}H NMR of 2a

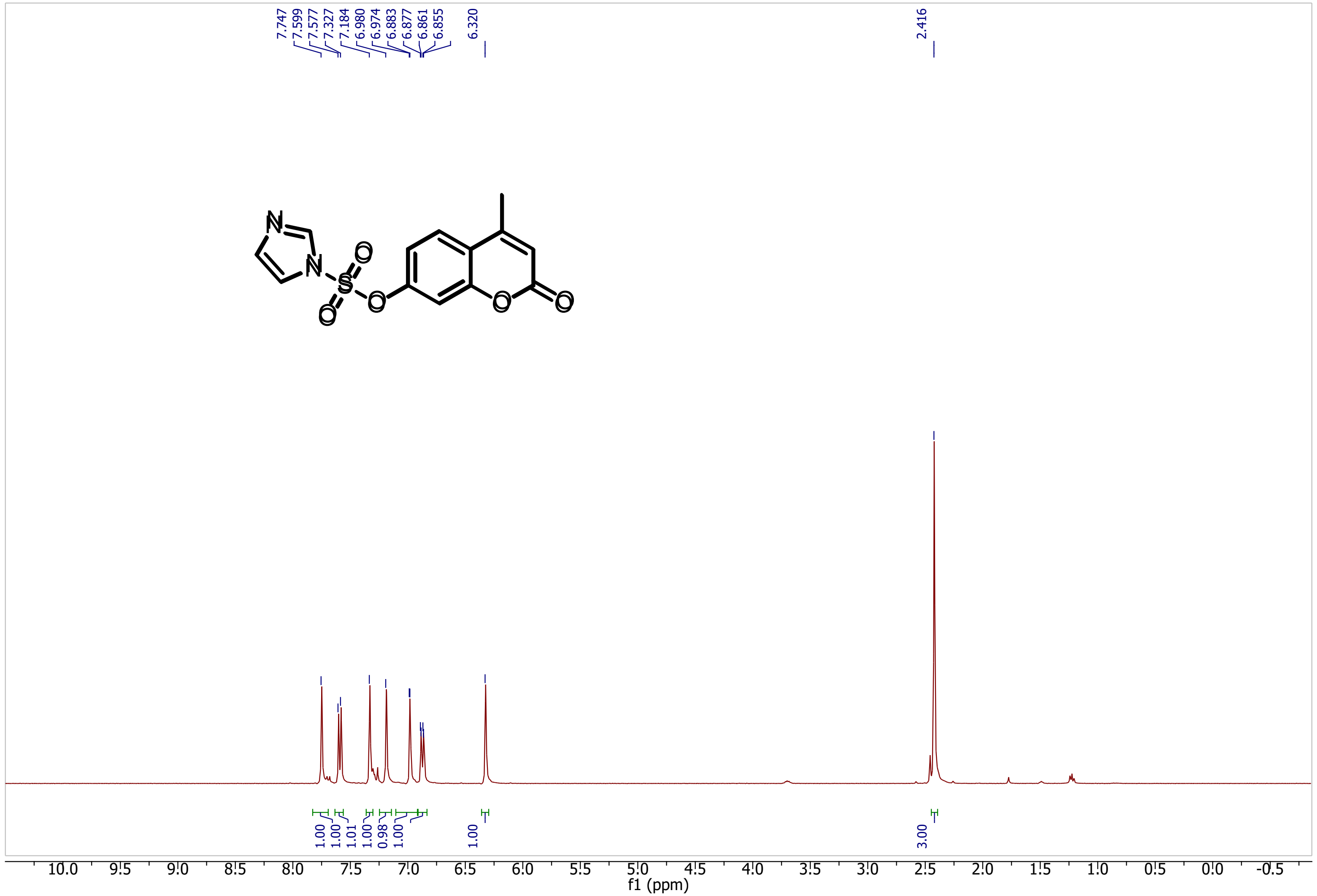

7.747
7.599
7.577
7.327
7.184
6.980
6.974
6.883
6.877
6.861
6.855
6.320
2.416
1.00
1.00
1.01
1.00
0.98
1.00
1.00
3.00
10.0
9.5
9.0
8.5
8.0
7.5
7.0
6.5
6.0
5.5
5.0
4.5
4.0
3.5
3.0
2.5
2.0
1.5
1.0
0.5
0.0
-0.5
f1 (ppm)

^{13}C NMR of 2a

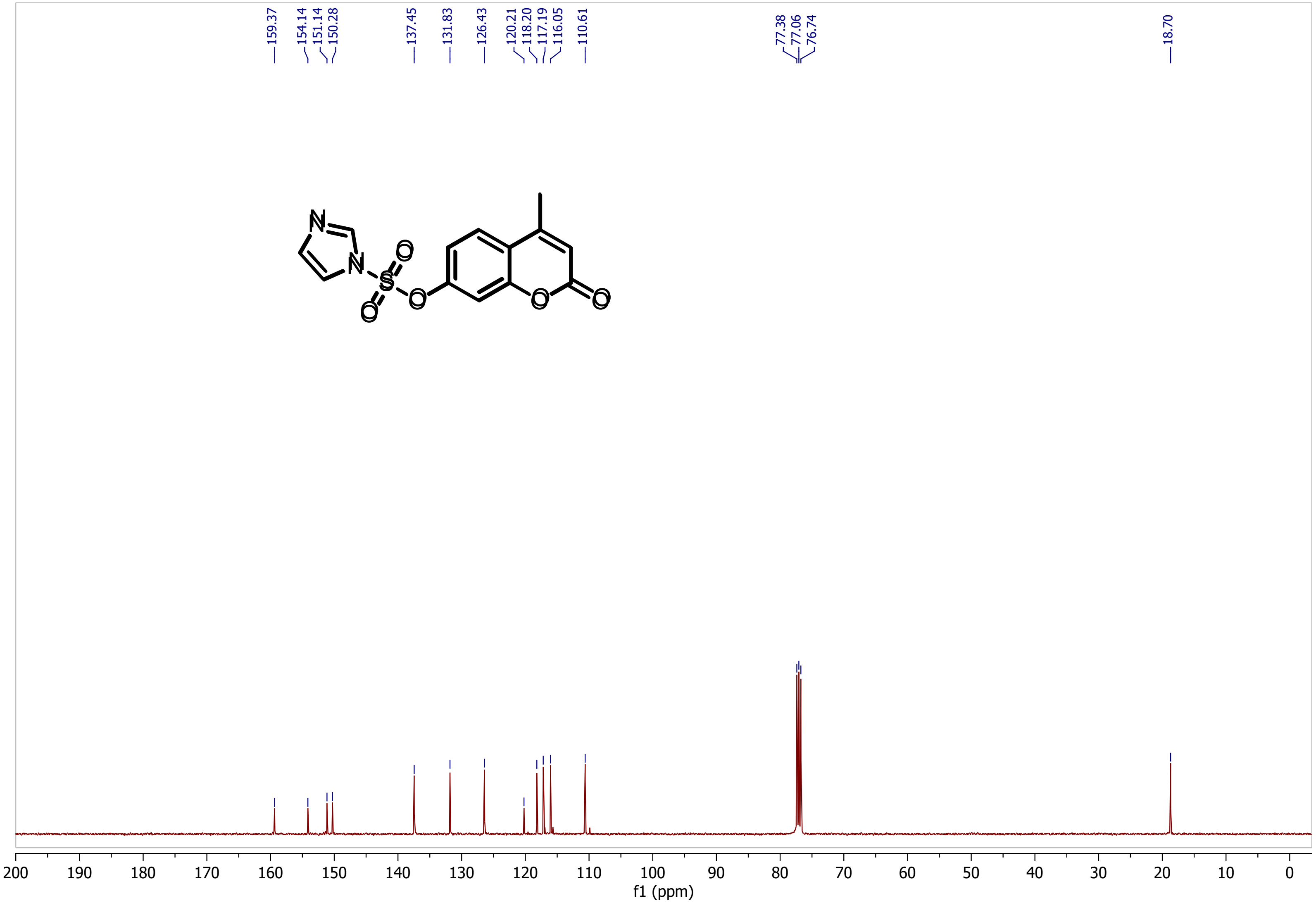

159.37
154.14
151.14
150.28
137.45
131.83
126.43
120.21
118.20
117.19
116.05
110.61
77.38
77.06
76.74
18.70
200
190
180
170
160
150
140
130
120
110
100
90
80
70
60
50
40
30
20
10
0
f1 (ppm)

^{1}H NMR of 4aa

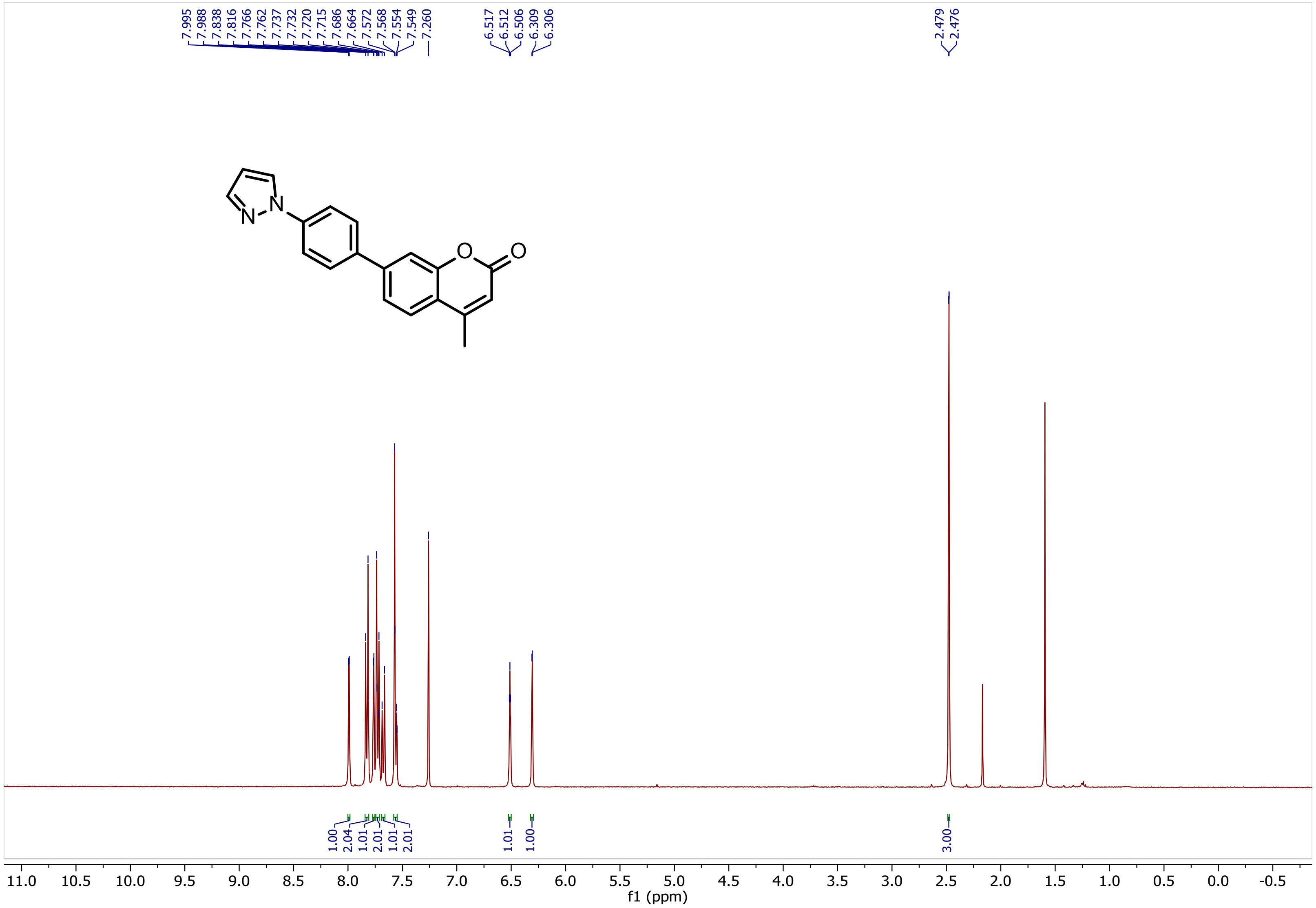

7.995
7.988
7.838
7.816
7.766
7.762
7.737
7.732
7.720
7.715
7.686
7.664
7.572
7.568
7.554
7.549
7.260
6.517
6.512
6.506
6.309
6.306
2.479
2.476
N
N
O
O
1.00
2.04
1.01
2.01
1.01
2.01
1.01
1.00
3.00
11.0
10.5
10.0
9.5
9.0
8.5
8.0
7.5
7.0
6.5
6.0
5.5
5.0
4.5
4.0
3.5
3.0
2.5
2.0
1.5
1.0
0.5
0.0
-0.5
f1 (ppm)

^{13}C NMR of 4aa

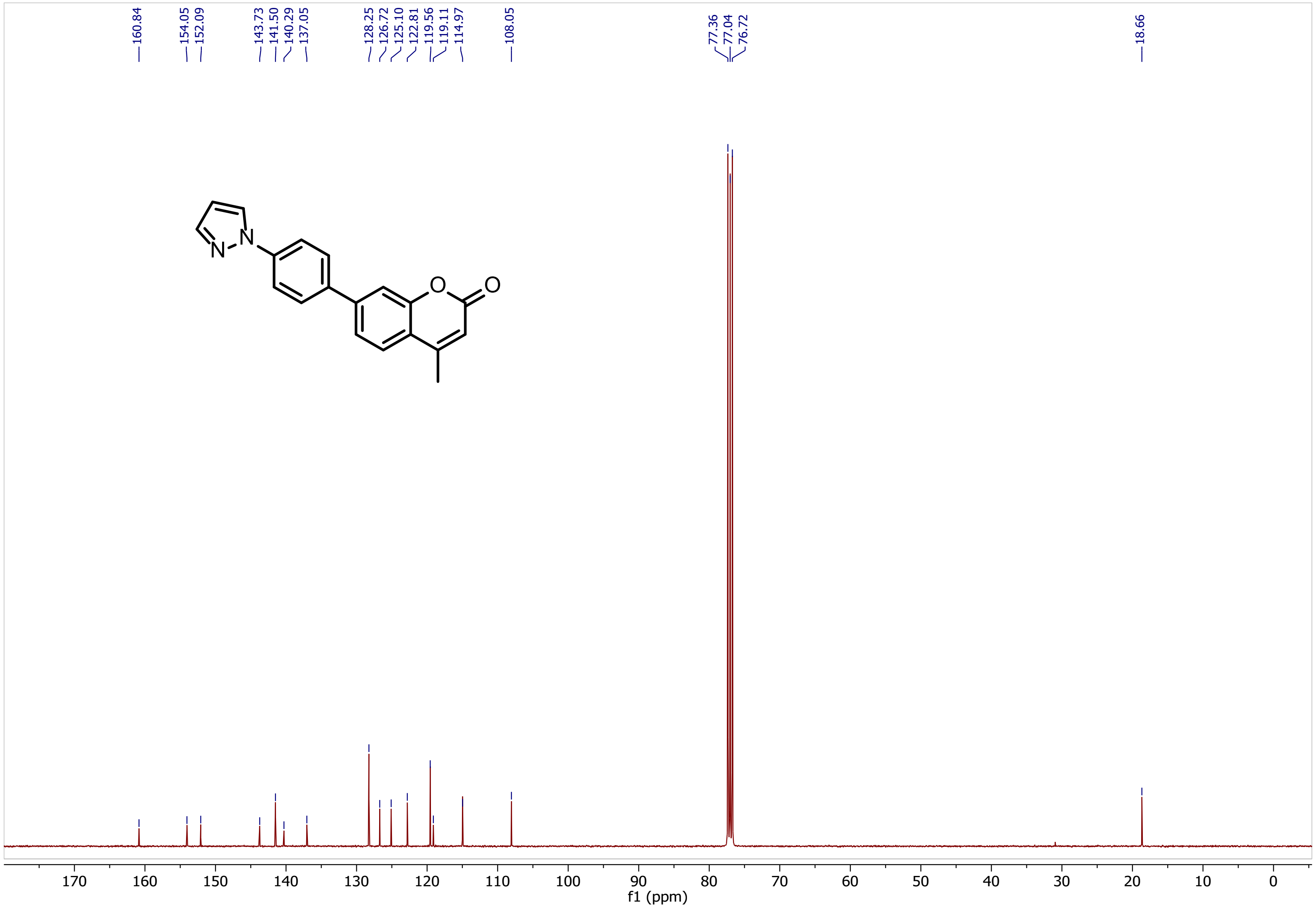

^{1}H NMR of 4ab

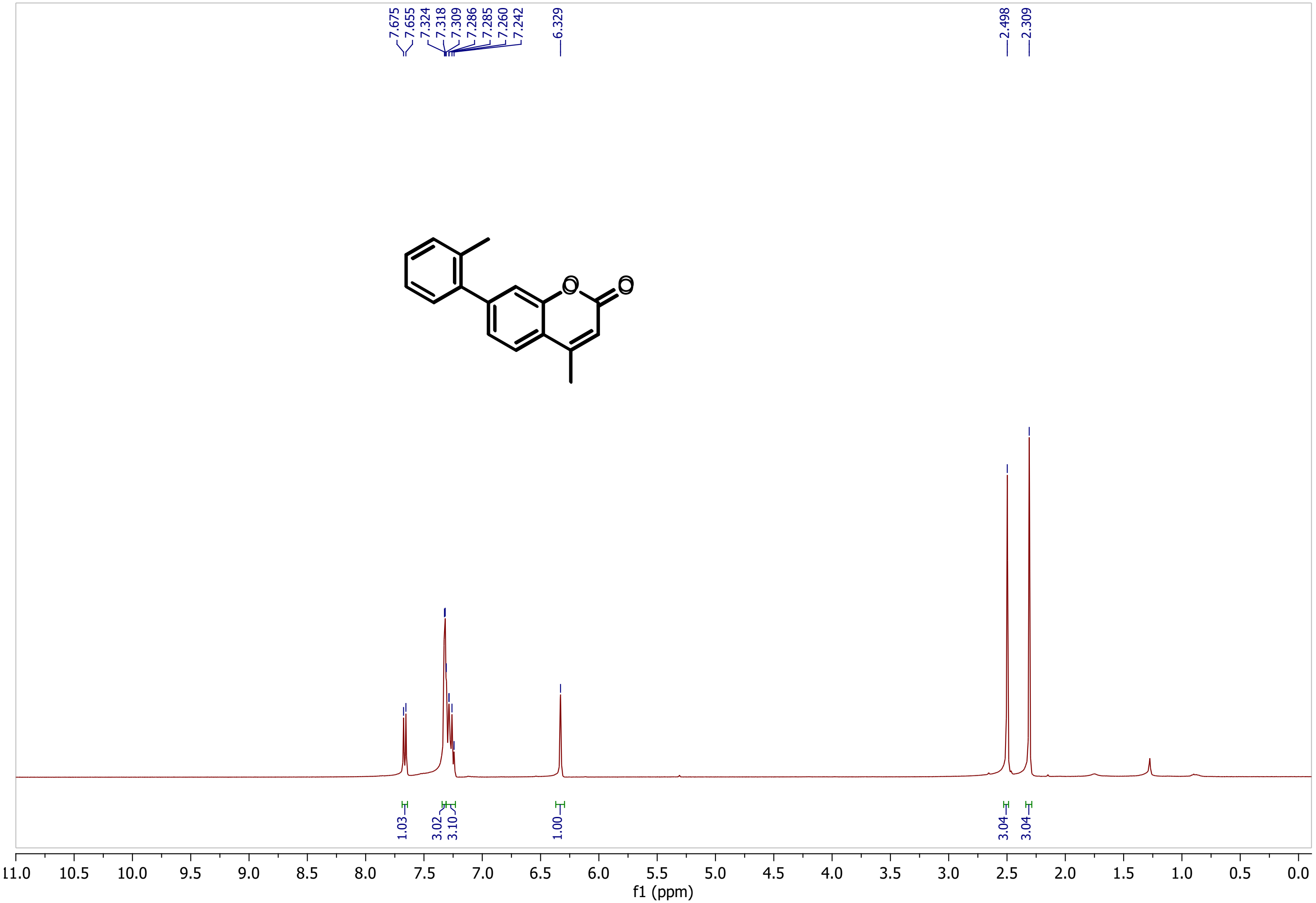
7.675
7.655
7.324
7.318
7.309
7.286
7.285
7.260
7.242
6.329
2.498
2.309
1.03
3.02
3.10
1.00
3.04
3.04
11.0 10.5 10.0 9.5 9.0 8.5 8.0 7.5 7.0 6.5 6.0 5.5 5.0 4.5 4.0 3.5 3.0 2.5 2.0 1.5 1.0 0.5 0.0
f1 (ppm)

^{13}C NMR of 4ab

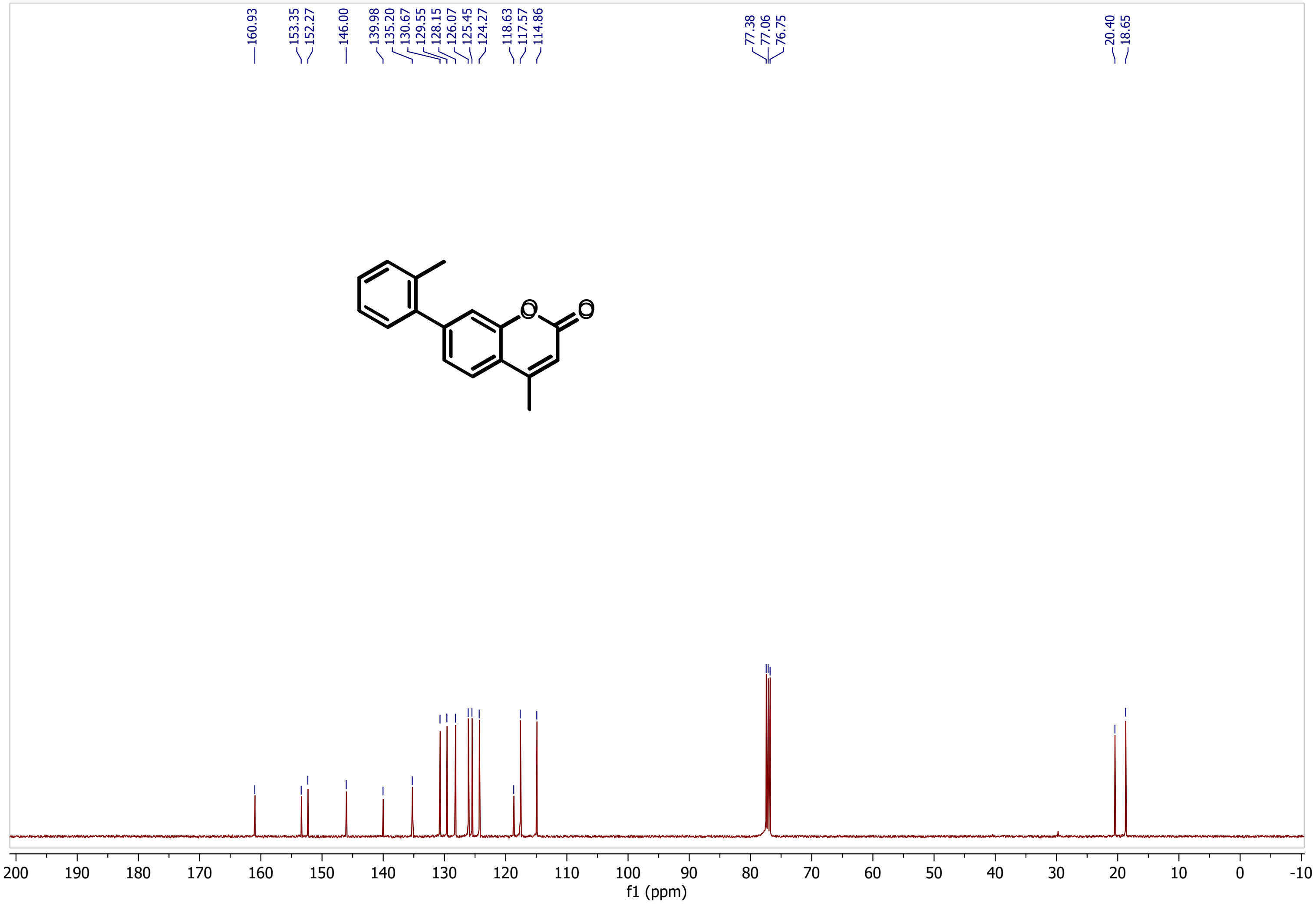

^{1}H NMR of 4ac

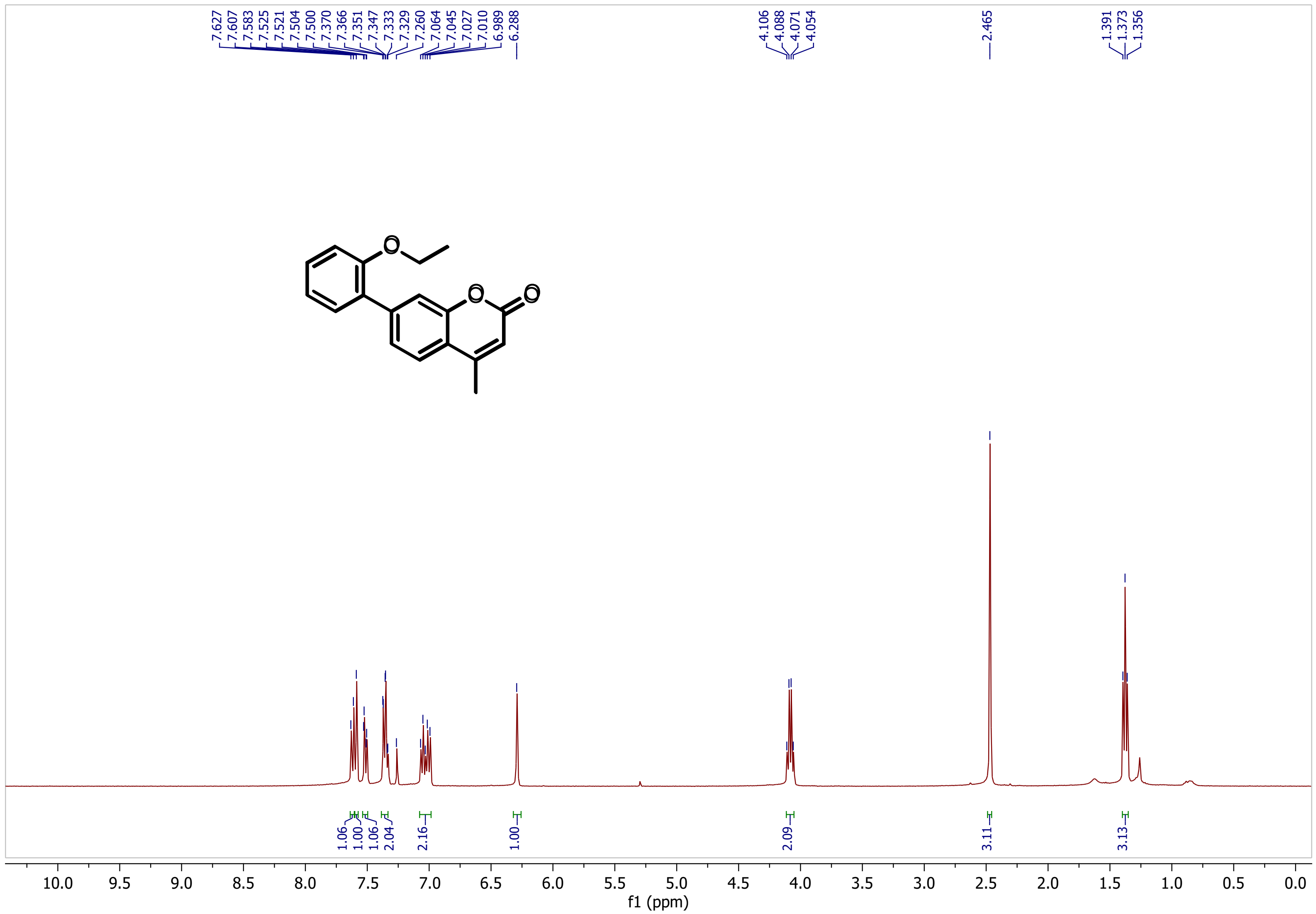
7.627
7.607
7.583
7.525
7.521
7.504
7.500
7.370
7.366
7.351
7.347
7.333
7.329
7.260
7.064
7.045
7.027
7.010
6.989
6.288
4.106
4.088
4.071
4.054
2.465
1.391
1.373
1.356
1.06
1.00
1.06
2.04
2.16
1.00
2.09
3.11
3.13
10.0 9.5 9.0 8.5 8.0 7.5 7.0 6.5 6.0 5.5 5.0 4.5 4.0 3.5 3.0 2.5 2.0 1.5 1.0 0.5 0.0
f1 (ppm)

^{13}C NMR of 4ac

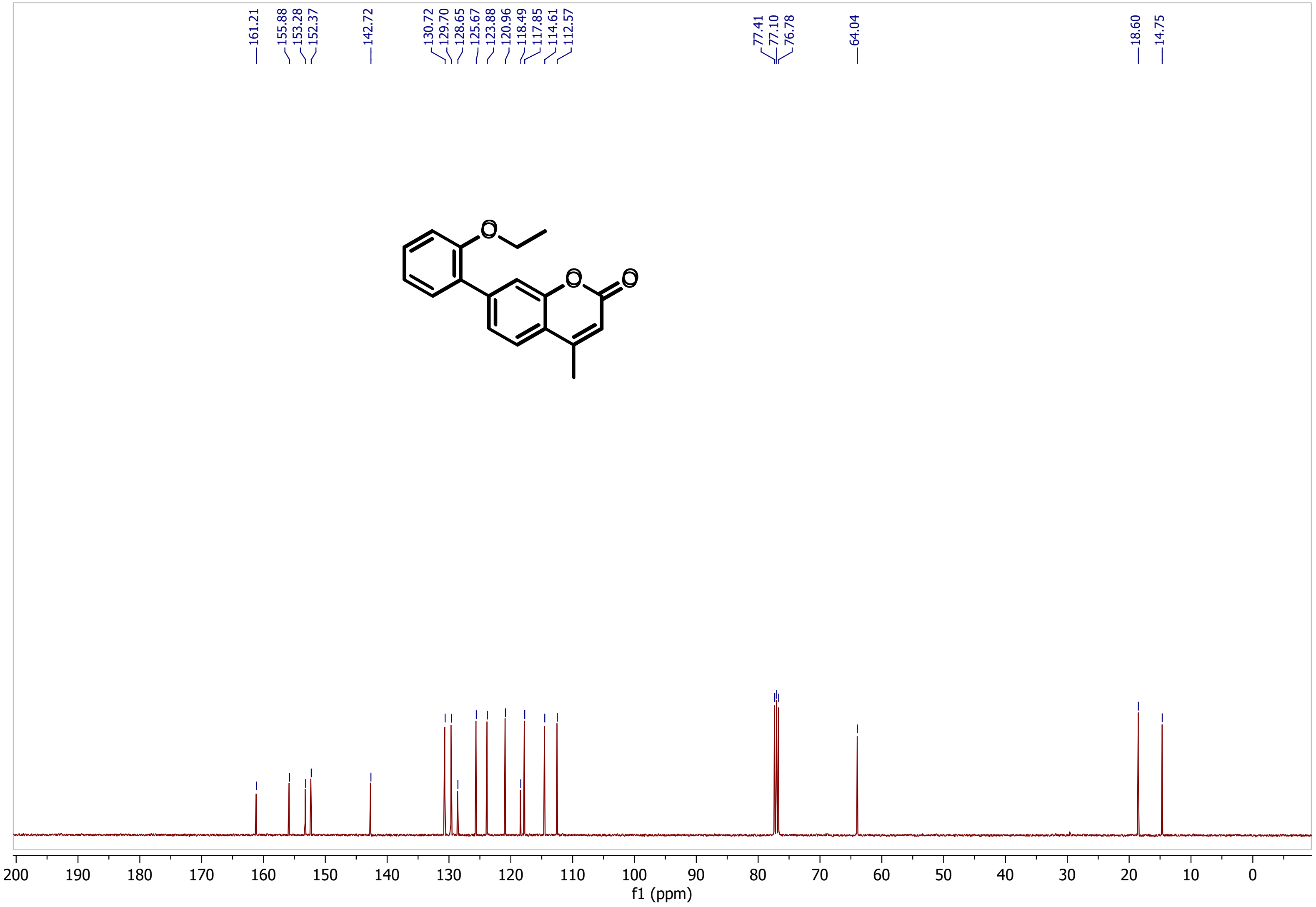

^{1}H NMR of 4ad

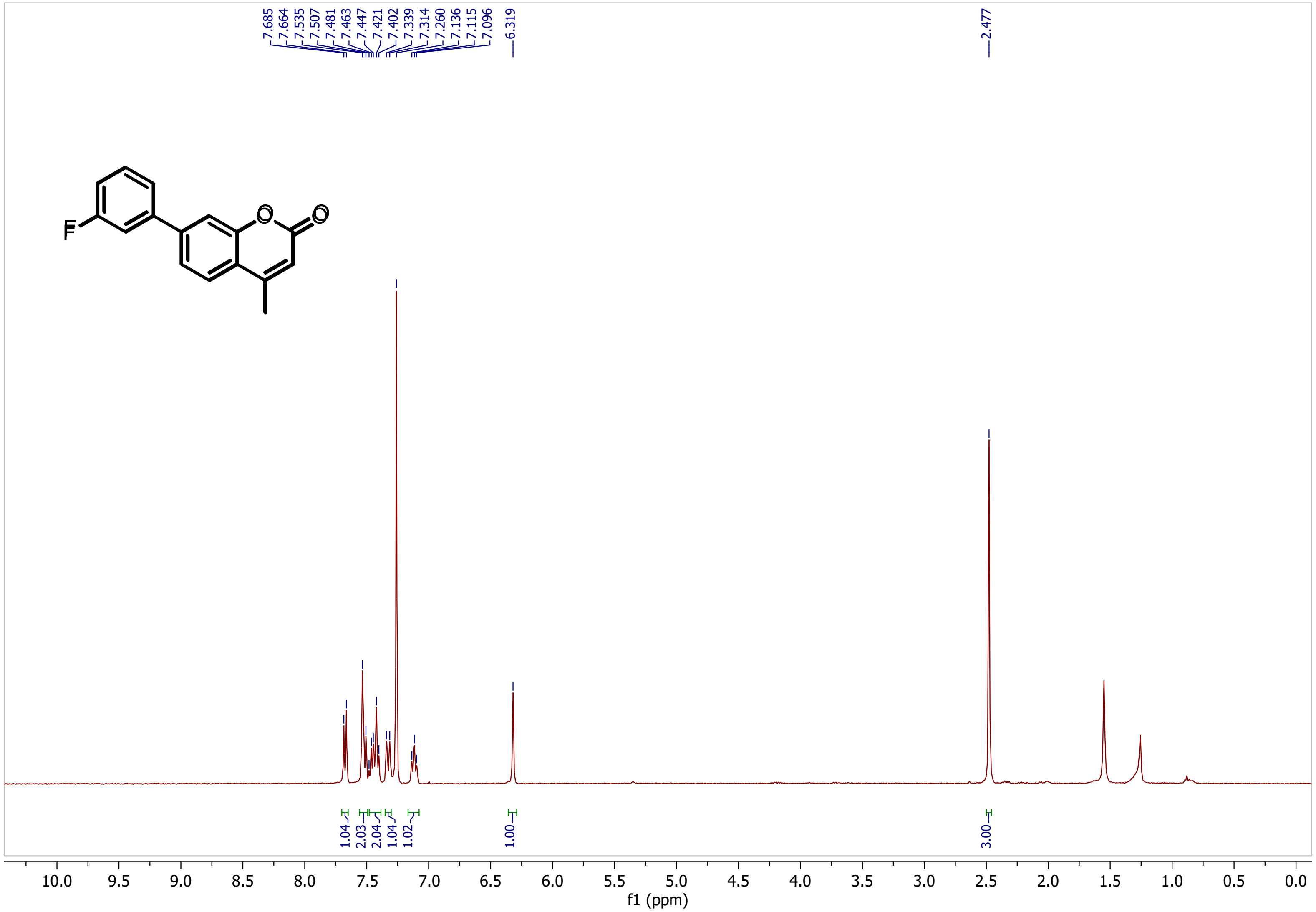
7.685
7.664
7.535
7.507
7.481
7.463
7.447
7.421
7.402
7.339
7.314
7.260
7.136
7.115
7.096
6.319
2.477
F
O
O
1.04
2.03
2.04
1.04
1.02
1.00
3.00
10.0
9.5
9.0
8.5
8.0
7.5
7.0
6.5
6.0
5.5
5.0
4.5
4.0
3.5
3.0
2.5
2.0
1.5
1.0
0.5
0.0
f1 (ppm)

^{13}C NMR of 4ad

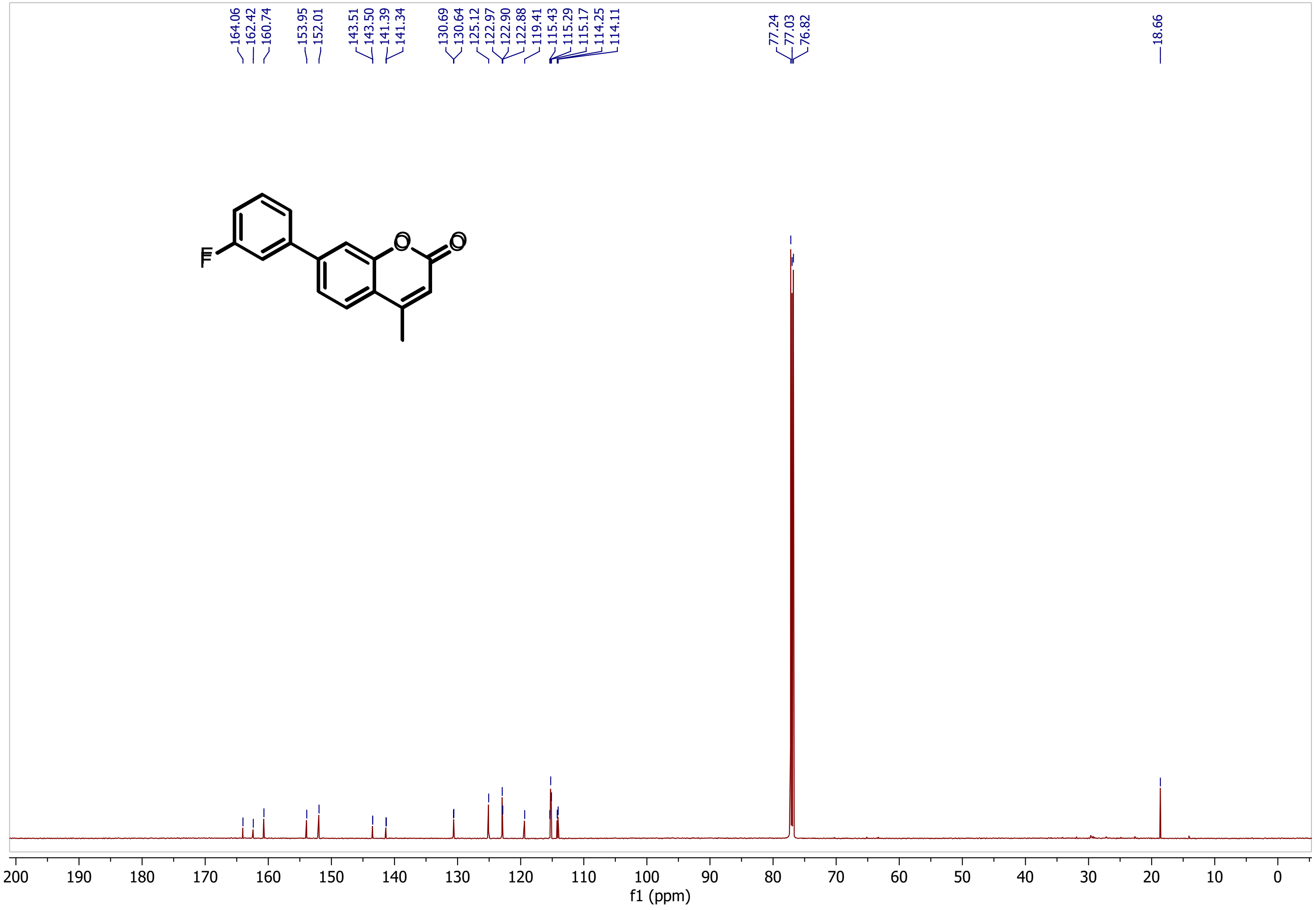

^{1}H NMR of 4ae

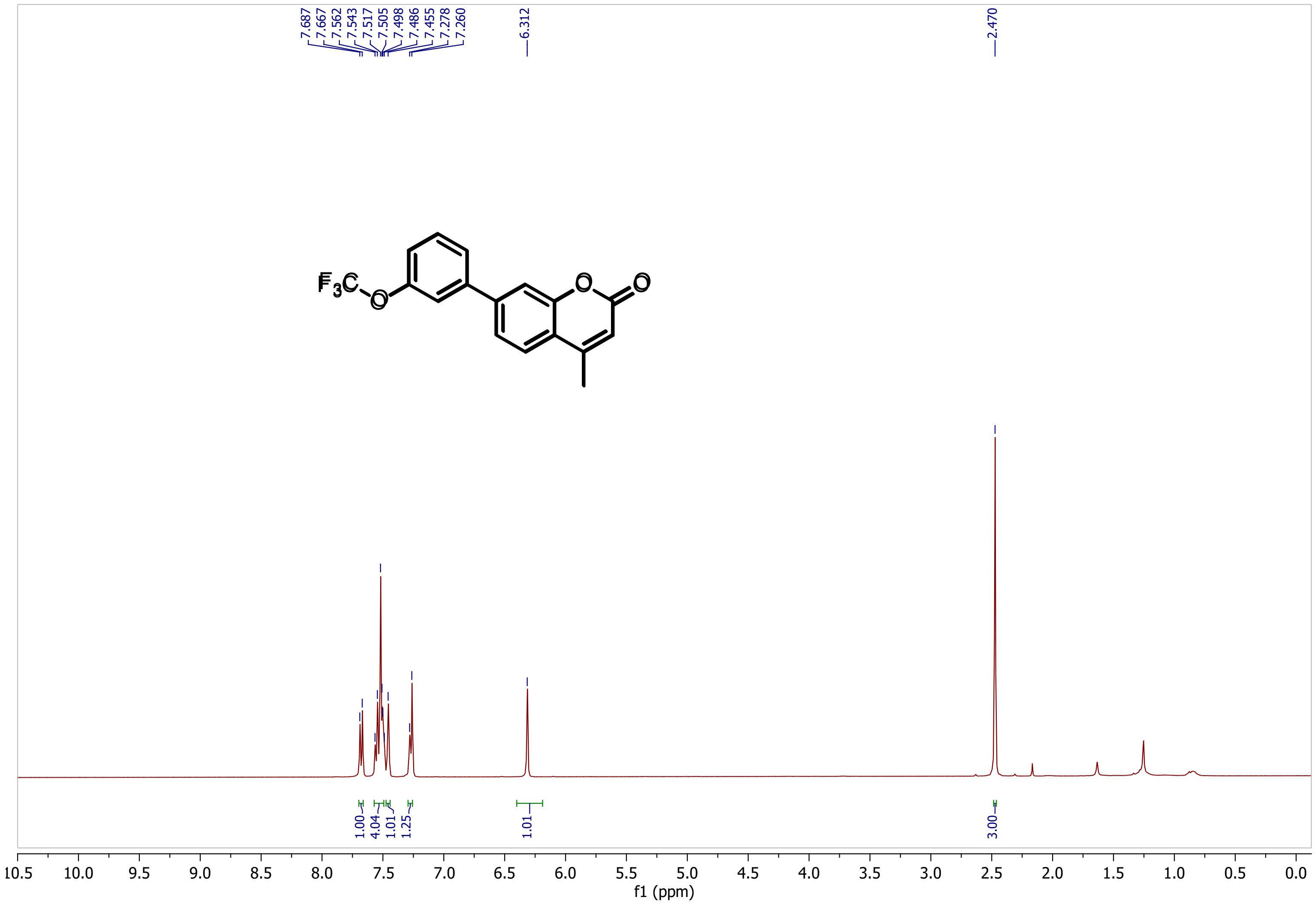
7.687
7.667
7.562
7.543
7.517
7.505
7.498
7.486
7.455
7.278
7.260
6.312
2.470
F3C
O
O
O
1.00
4.04
1.01
1.25
1.01
3.00
10.5 10.0 9.5 9.0 8.5 8.0 7.5 7.0 6.5 6.0 5.5 5.0 4.5 4.0 3.5 3.0 2.5 2.0 1.5 1.0 0.5 0.0
f1 (ppm)

^{13}C NMR of 4ae

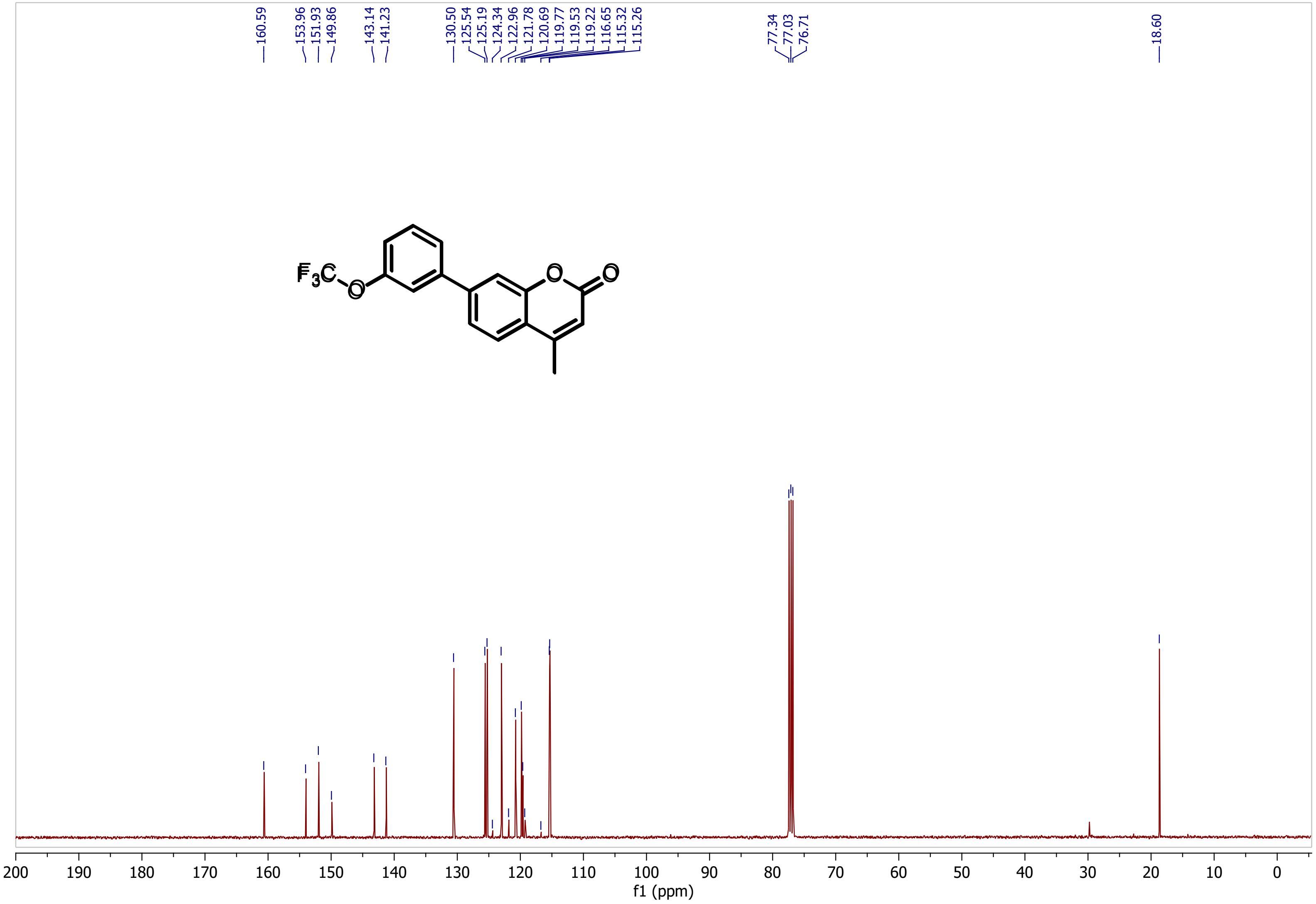

^{1}H NMR of 4af

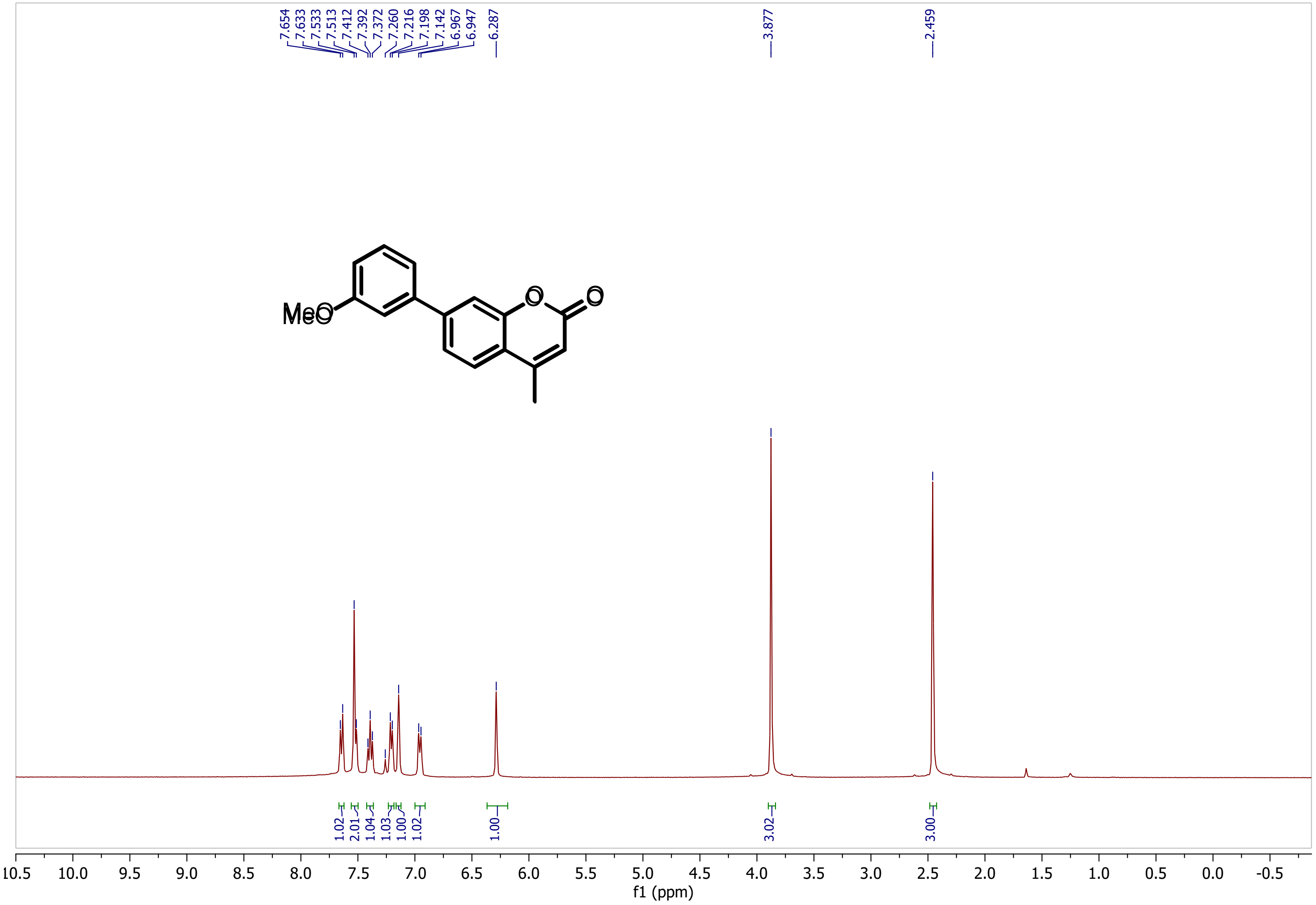
7.654
7.633
7.533
7.513
7.412
7.392
7.372
7.260
7.216
7.198
7.142
6.967
6.947
6.287
3.877
2.459
MeO
O
O
1.02
2.01
1.04
1.03
1.00
1.02
1.00
3.02
3.00
10.5
10.0
9.5
9.0
8.5
8.0
7.5
7.0
6.5
6.0
5.5
5.0
4.5
4.0
3.5
3.0
2.5
2.0
1.5
1.0
0.5
0.0
-0.5
f1 (ppm)

^{13}C NMR of 4af

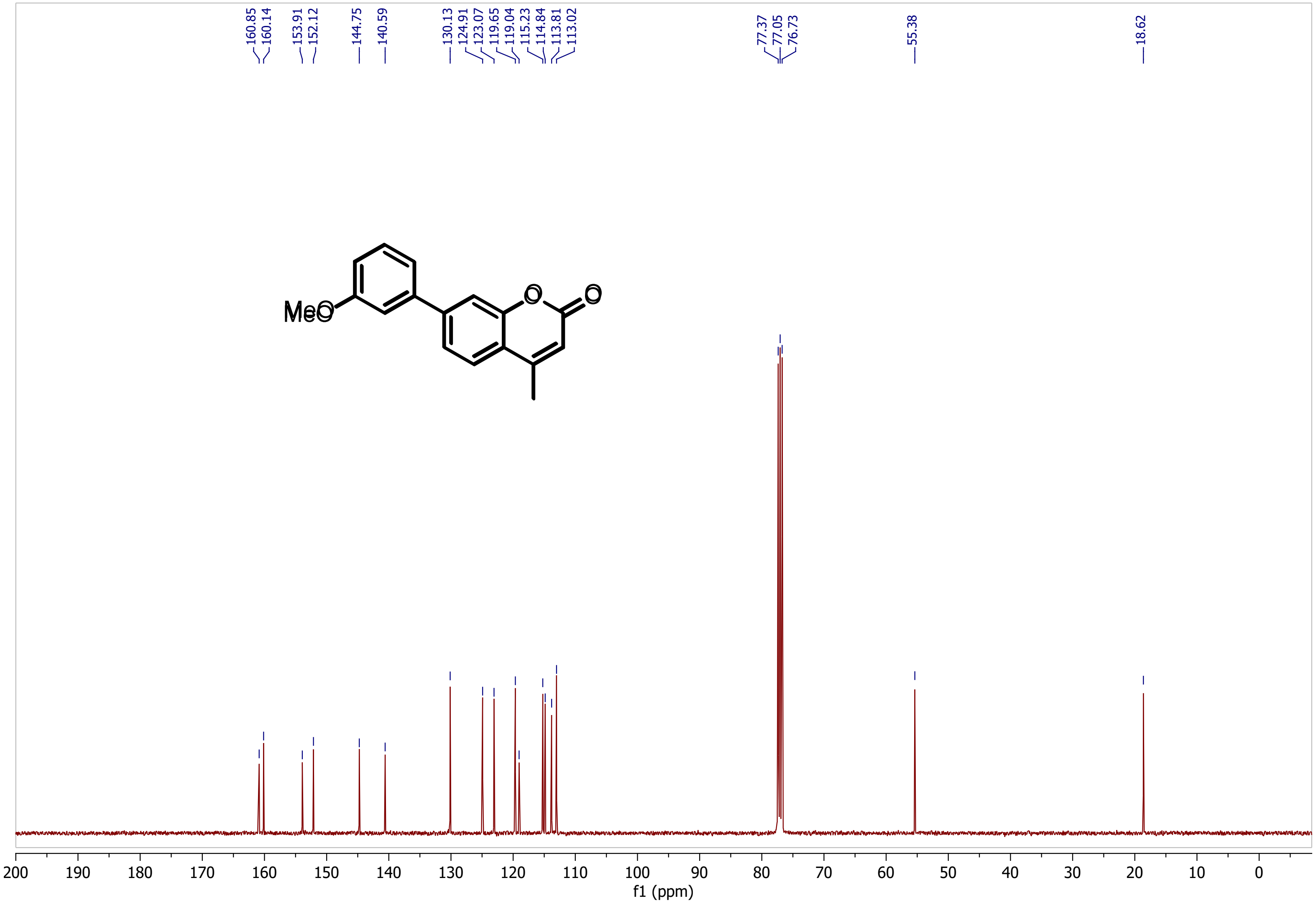

^{1}H NMR of 4ag

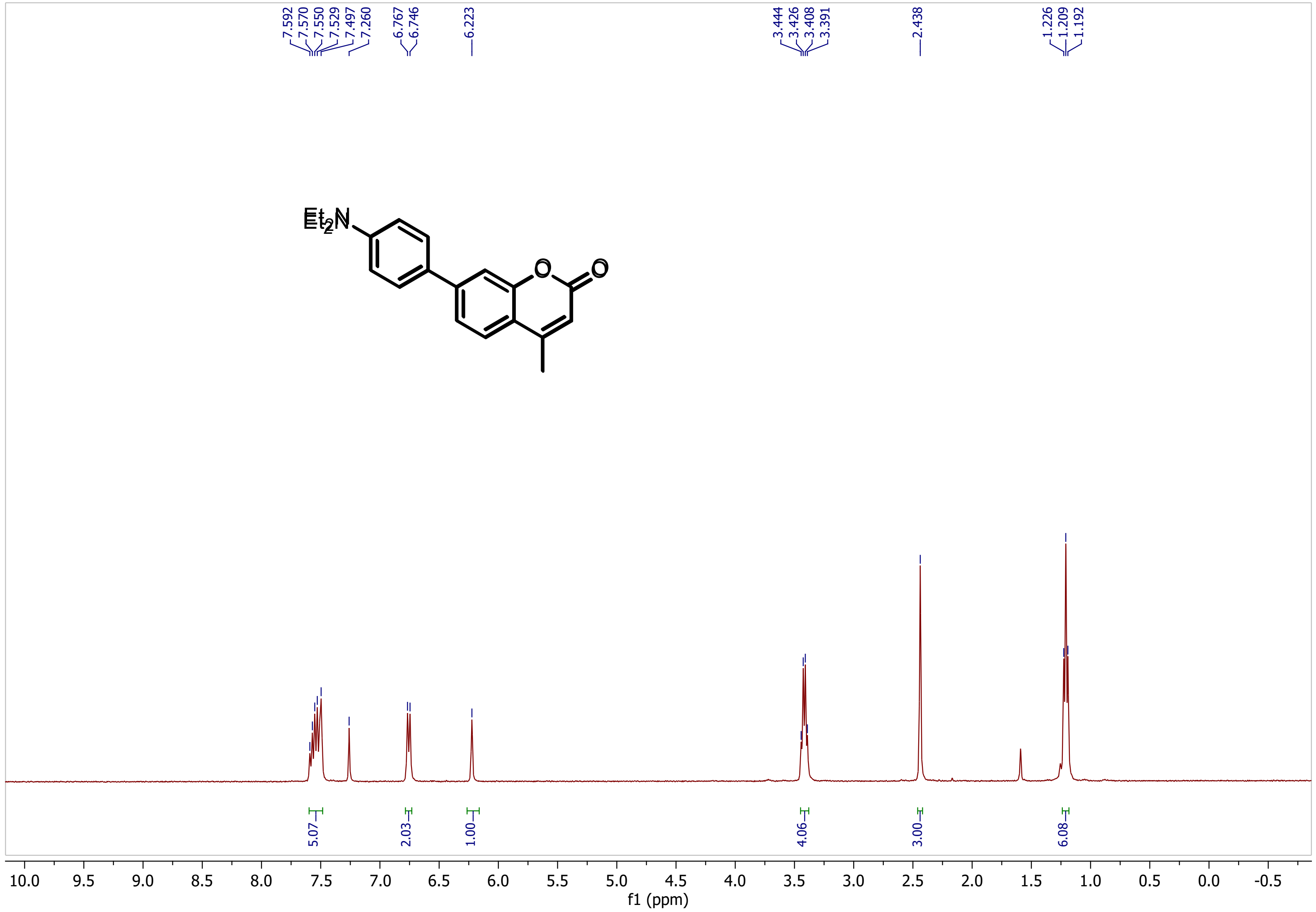
7.592
7.570
7.550
7.529
7.497
7.260
6.767
6.746
6.223
3.444
3.426
3.408
3.391
2.438
1.226
1.209
1.192
Et2N
O
O
5.07
2.03
1.00
4.06
3.00
6.08
10.0 9.5 9.0 8.5 8.0 7.5 7.0 6.5 6.0 5.5 5.0 4.5 4.0 3.5 3.0 2.5 2.0 1.5 1.0 0.5 0.0 -0.5
f1 (ppm)

^{13}C NMR of 4ag

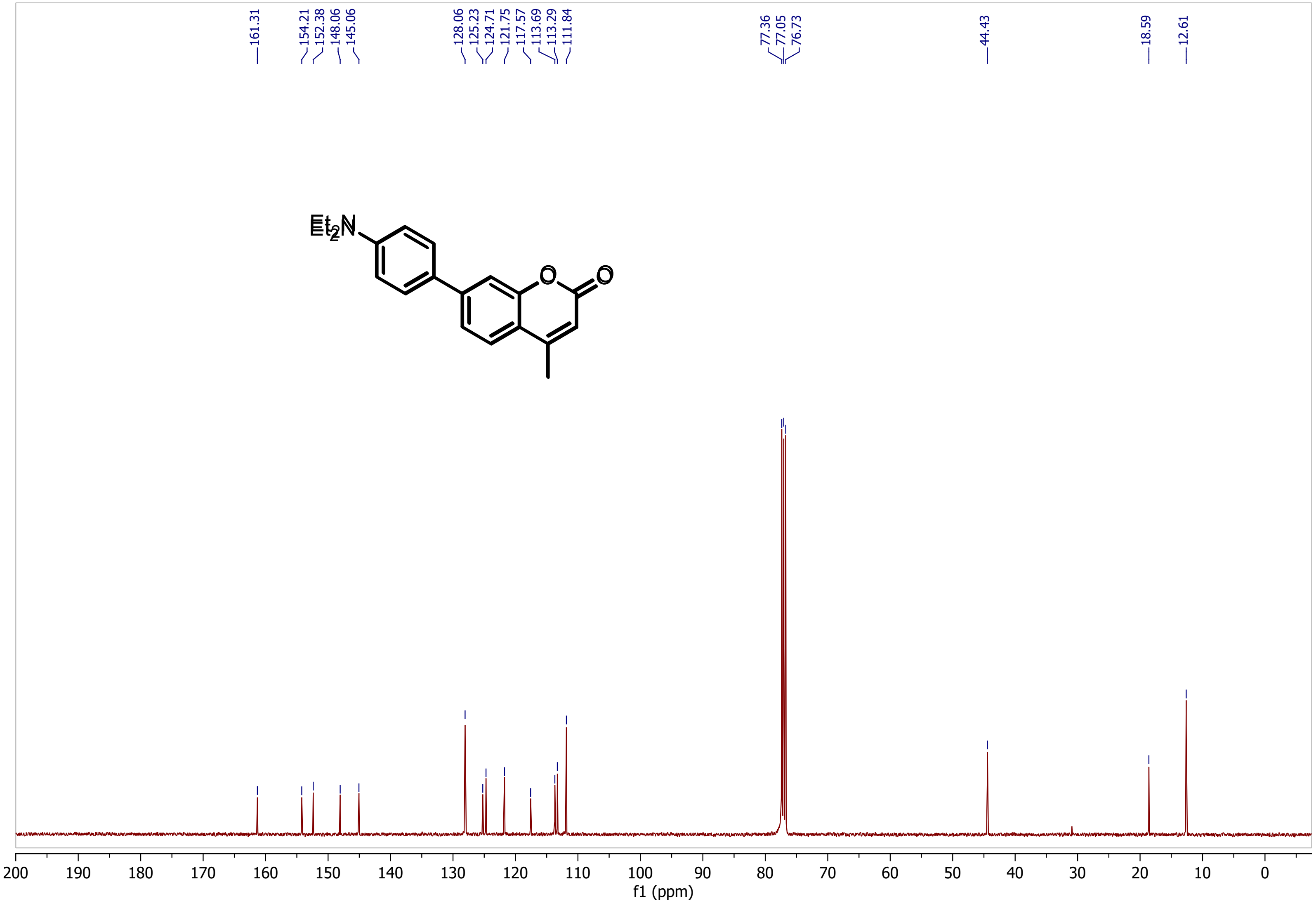

^{1}H NMR of 4ah

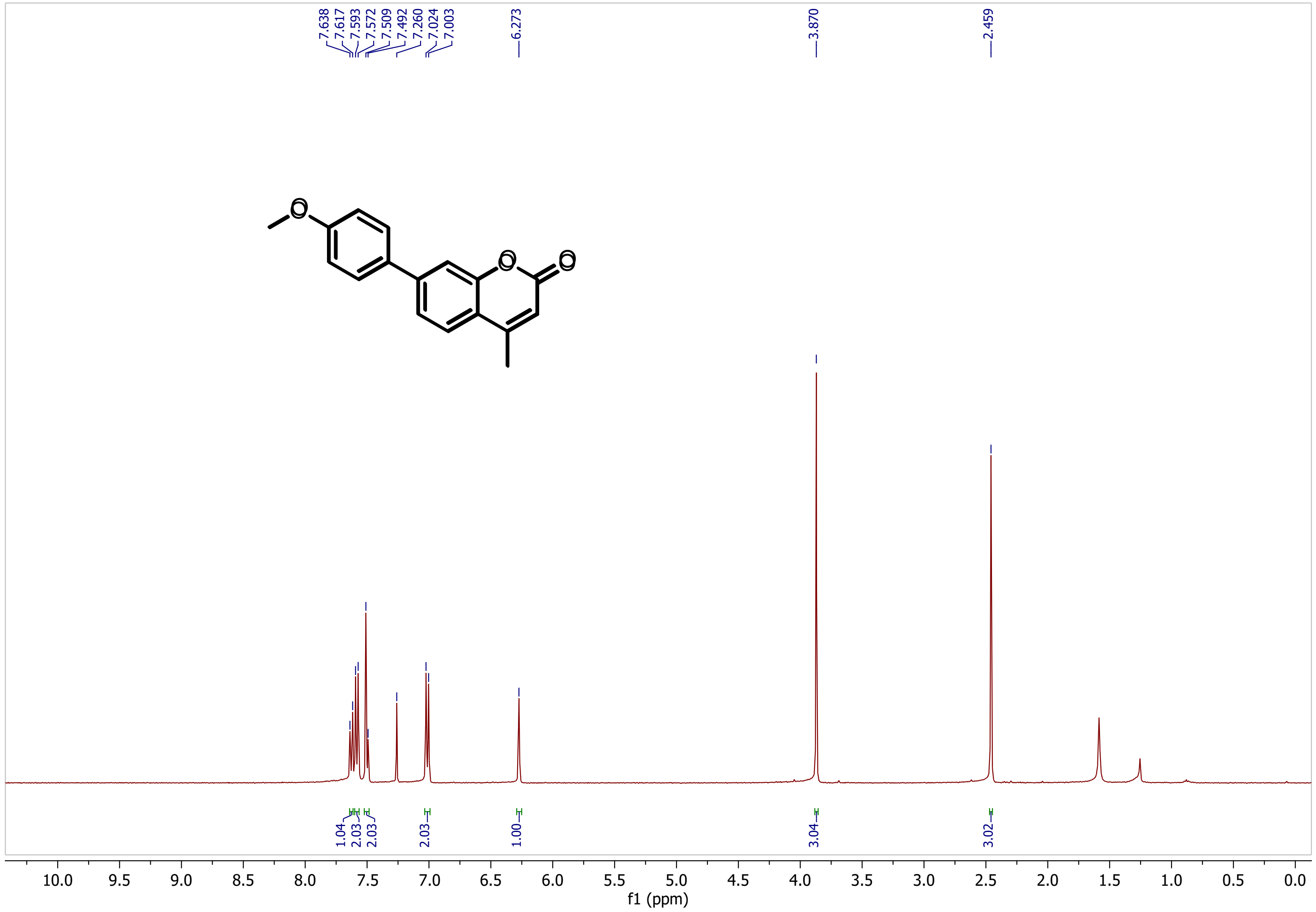
7.638
7.617
7.593
7.572
7.509
7.492
7.260
7.024
7.003
6.273
3.870
2.459
1.04
2.03
2.03
2.03
1.00
3.04
3.02
10.0
9.5
9.0
8.5
8.0
7.5
7.0
6.5
6.0
5.5
5.0
4.5
4.0
3.5
3.0
2.5
2.0
1.5
1.0
0.5
0.0
f1 (ppm)

^{13}C NMR of 4ah

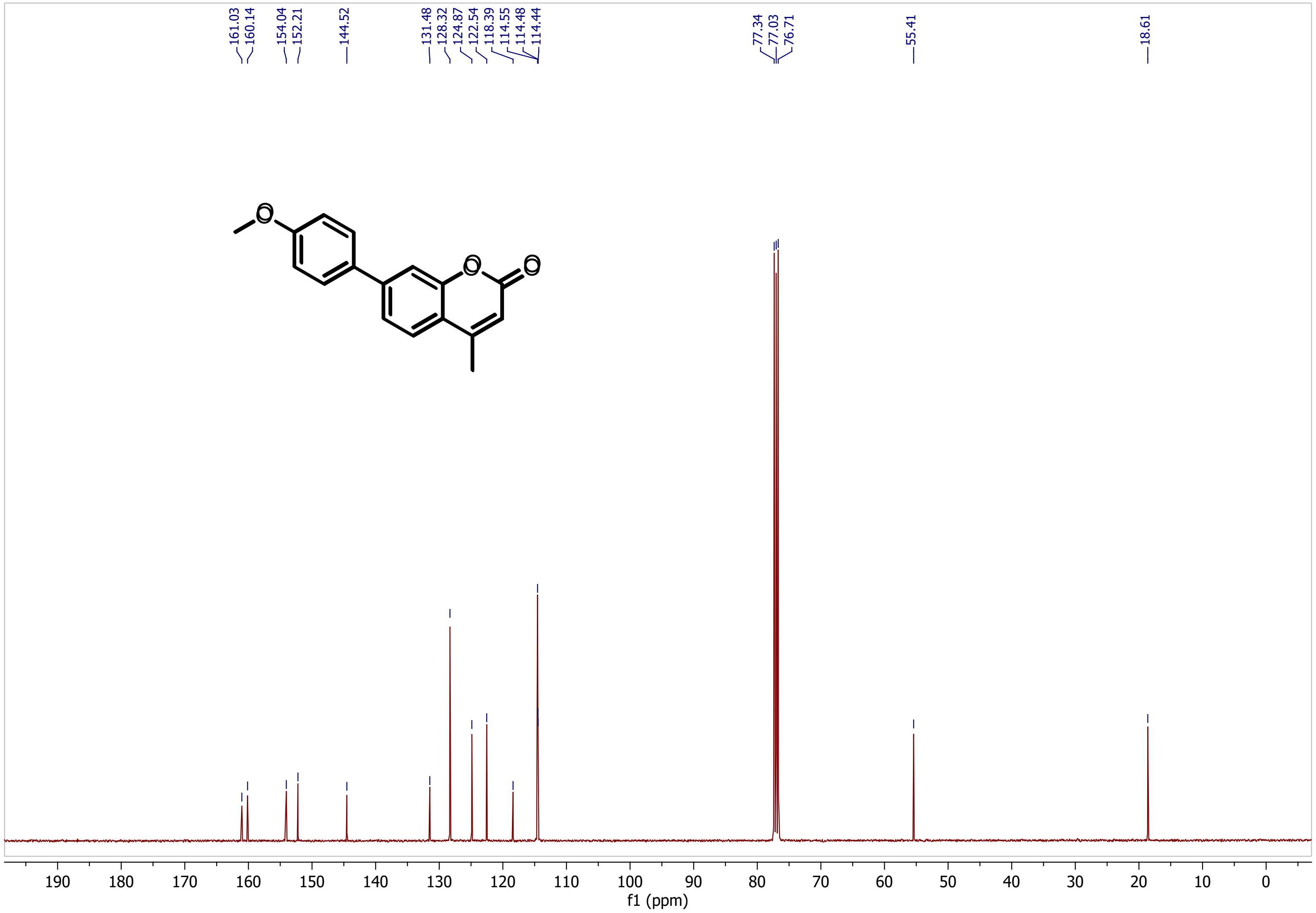

^{1}H NMR of 4bh

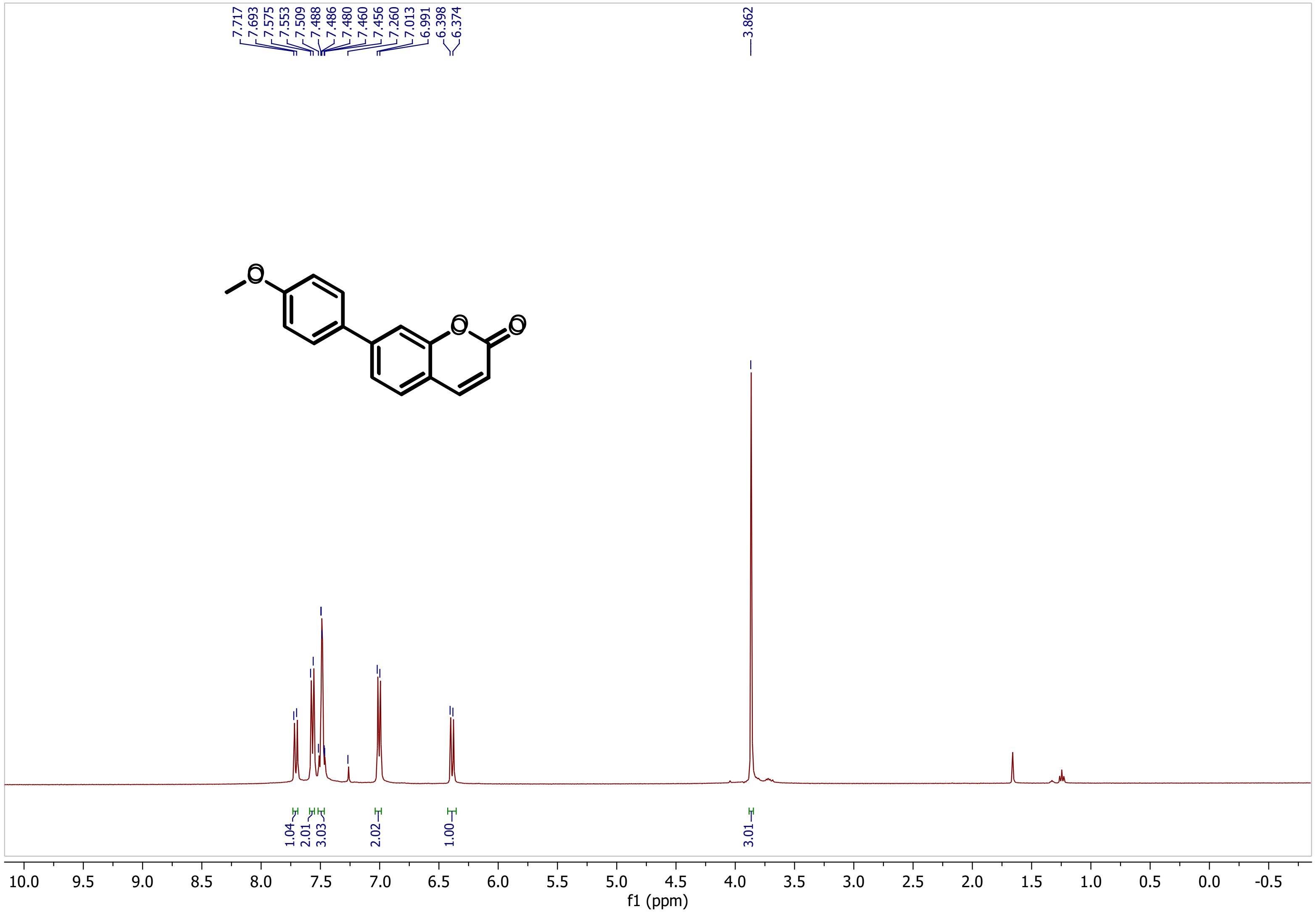

7.717
7.693
7.575
7.553
7.509
7.488
7.486
7.480
7.460
7.456
7.260
7.013
6.991
6.398
6.374
3.862
1.04
2.01
3.03
2.02
1.00
3.01
10.0 9.5 9.0 8.5 8.0 7.5 7.0 6.5 6.0 5.5 5.0 4.5 4.0 3.5 3.0 2.5 2.0 1.5 1.0 0.5 0.0 -0.5
f1 (ppm)

^{13}C NMR of 4bh

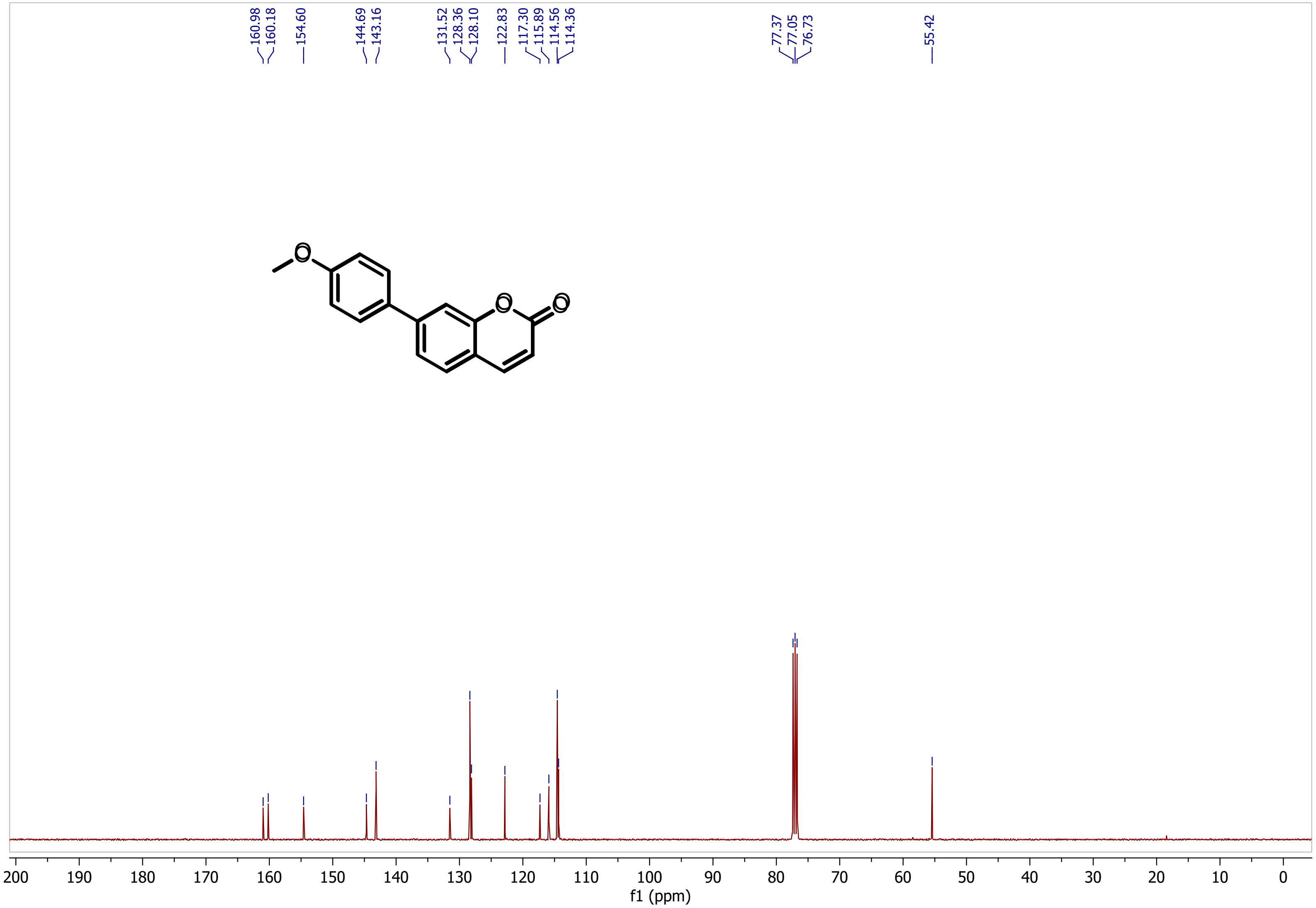

^{1}H NMR of 4ch

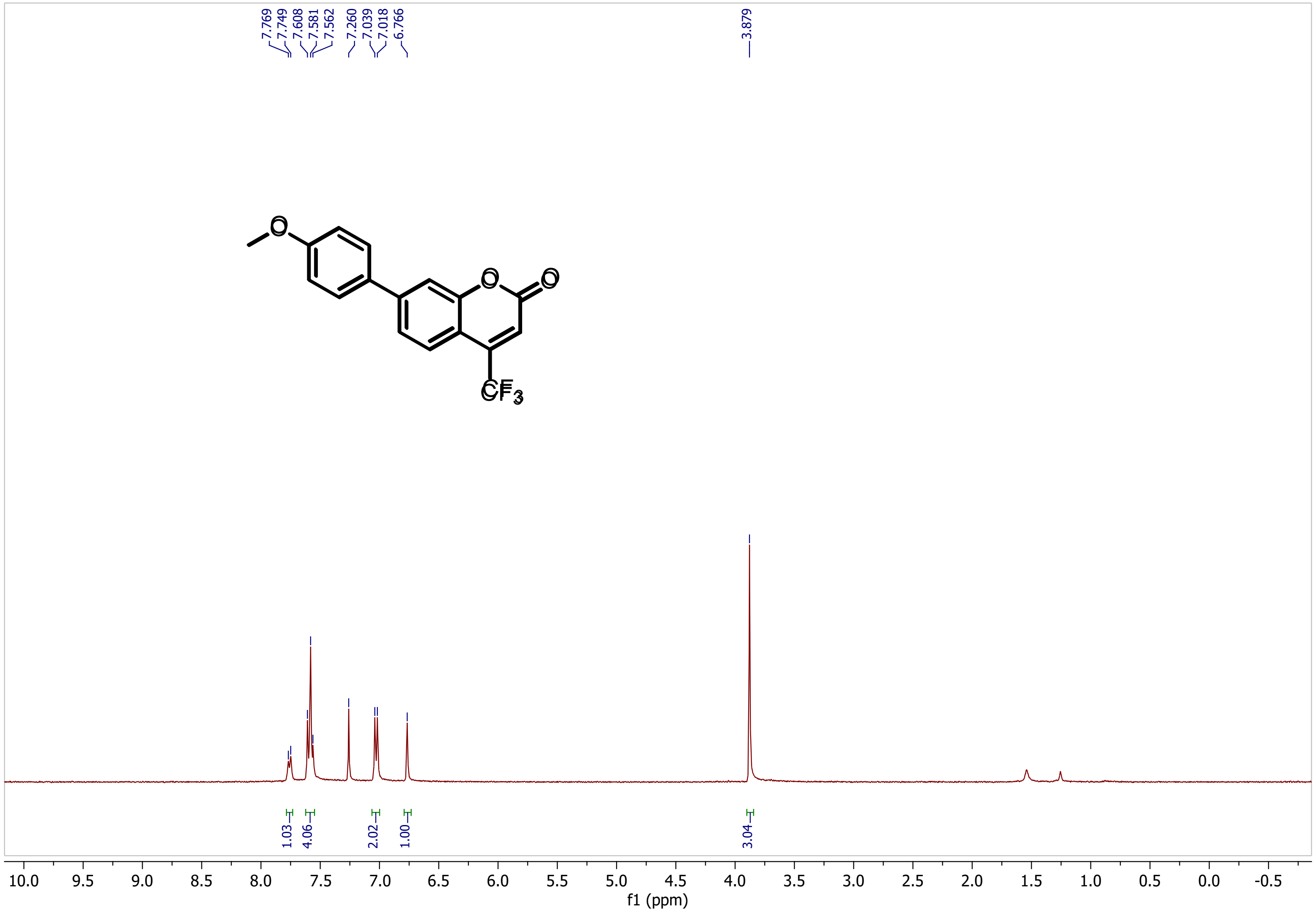

7.769
7.749
7.608
7.581
7.562
7.260
7.039
7.018
6.766
3.879
O
O
O
CF3
1.03
4.06
2.02
1.00
3.04
10.0 9.5 9.0 8.5 8.0 7.5 7.0 6.5 6.0 5.5 5.0 4.5 4.0 3.5 3.0 2.5 2.0 1.5 1.0 0.5 0.0 -0.5
f1 (ppm)

^{13}C NMR of 4ch

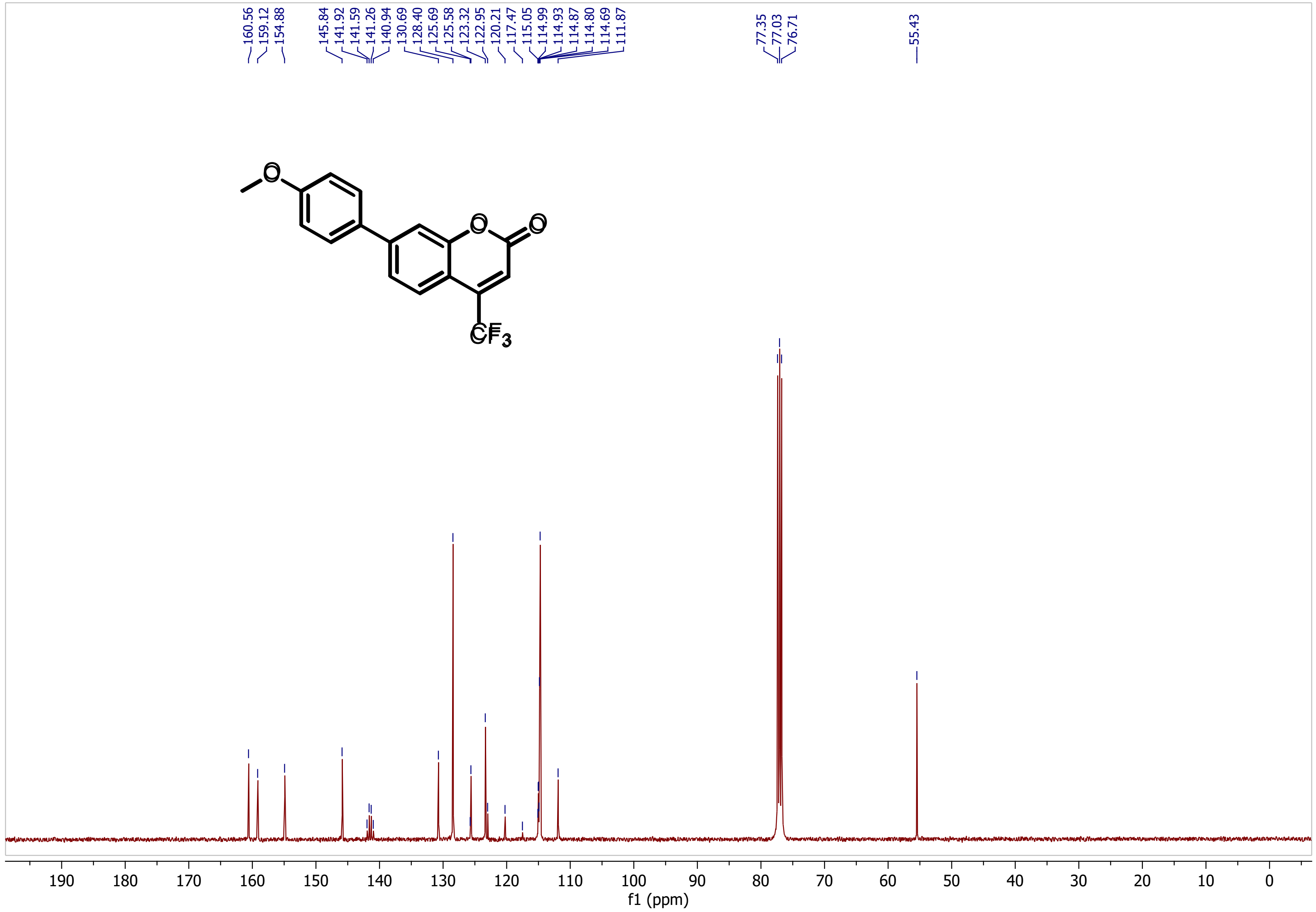

^{1}H NMR of 4dh

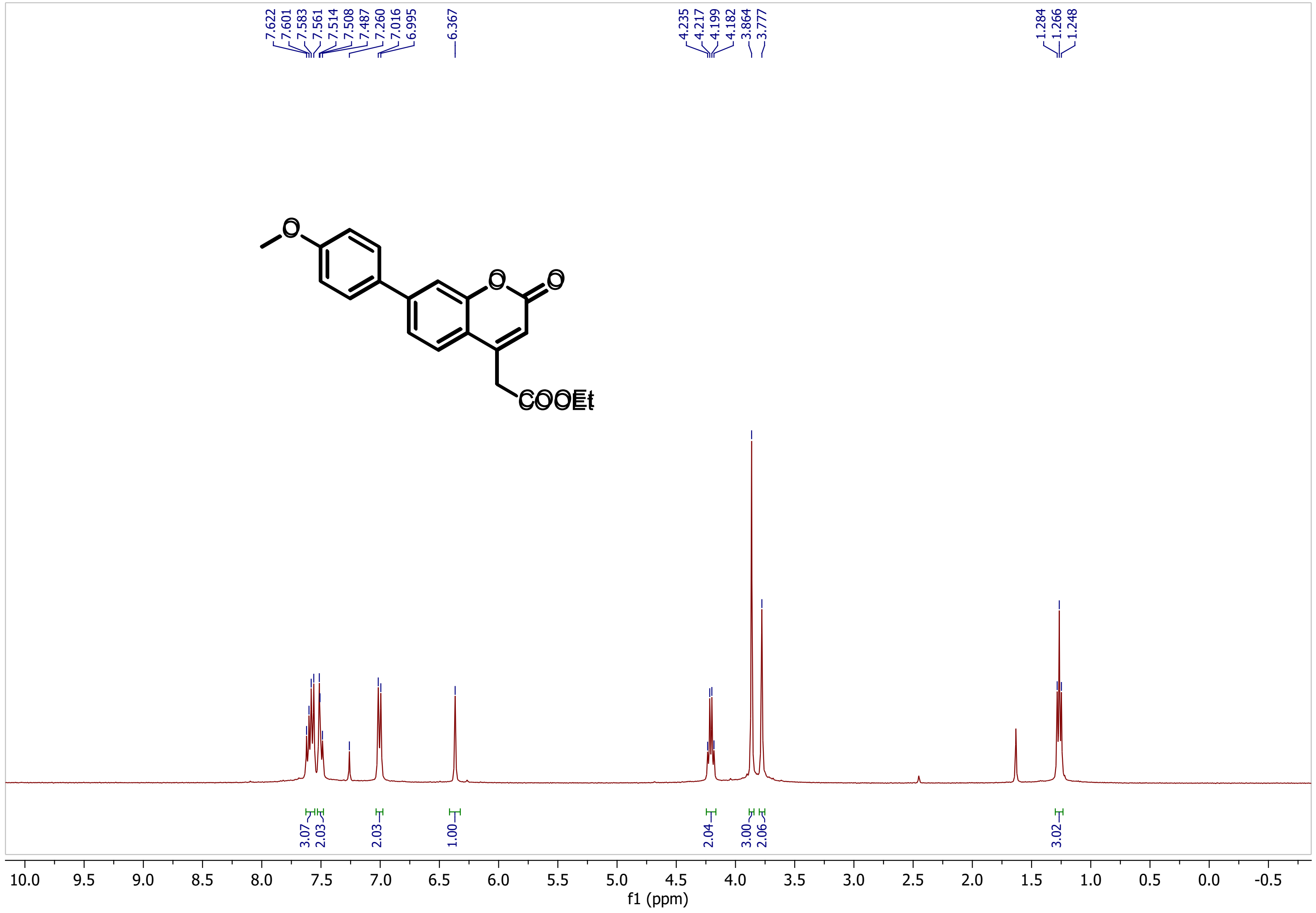

7.622
7.601
7.583
7.561
7.514
7.508
7.487
7.260
7.016
6.995
6.367
4.235
4.217
4.199
4.182
3.864
3.777
1.284
1.266
1.248
O
O
O
COOEt
3.07
2.03
2.03
1.00
2.04
3.00
2.06
3.02
10.0
9.5
9.0
8.5
8.0
7.5
7.0
6.5
6.0
5.5
5.0
4.5
4.0
3.5
3.0
2.5
2.0
1.5
1.0
0.5
0.0
-0.5
f1 (ppm)

^{13}C NMR of 4dh

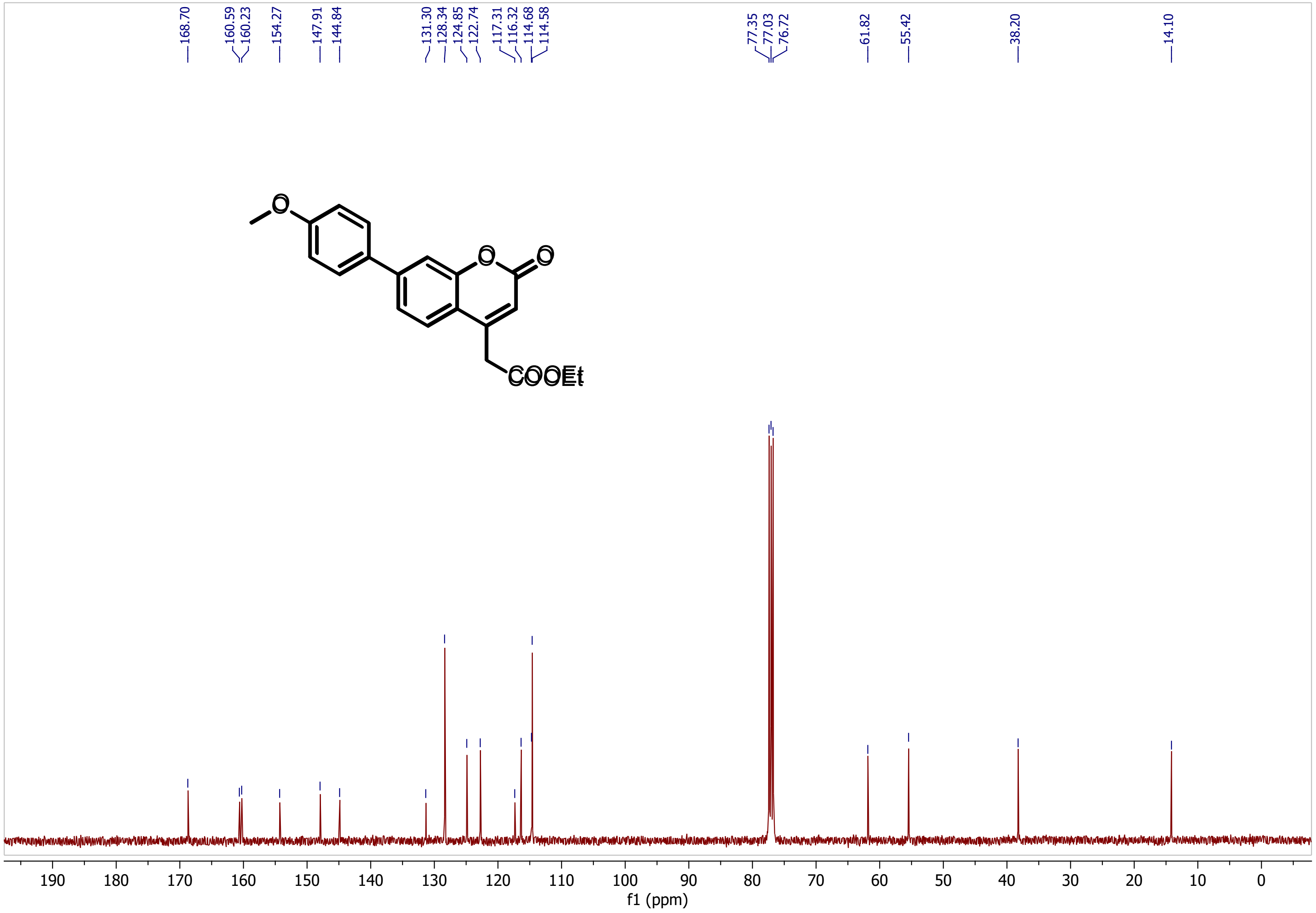

^{1}H NMR of 4eh

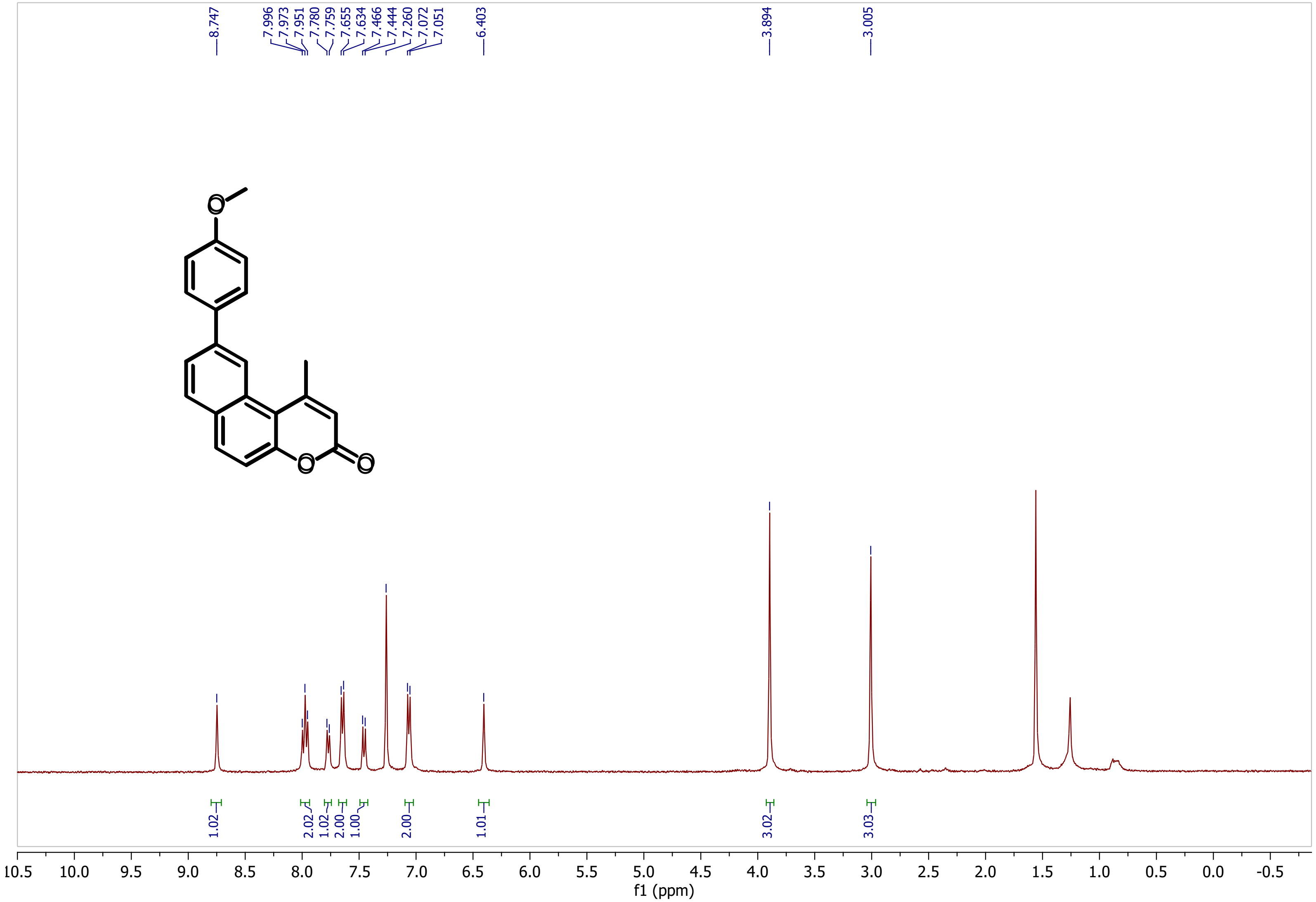
8.747
7.996
7.973
7.951
7.780
7.759
7.655
7.634
7.466
7.444
7.260
7.072
7.051
6.403
3.894
3.005
1.02
2.02
1.02
2.00
1.00
2.00
1.01
3.02
3.03
10.5
10.0
9.5
9.0
8.5
8.0
7.5
7.0
6.5
6.0
5.5
5.0
4.5
4.0
3.5
3.0
2.5
2.0
1.5
1.0
0.5
0.0
-0.5
f1 (ppm)

^{13}C NMR of 4eh

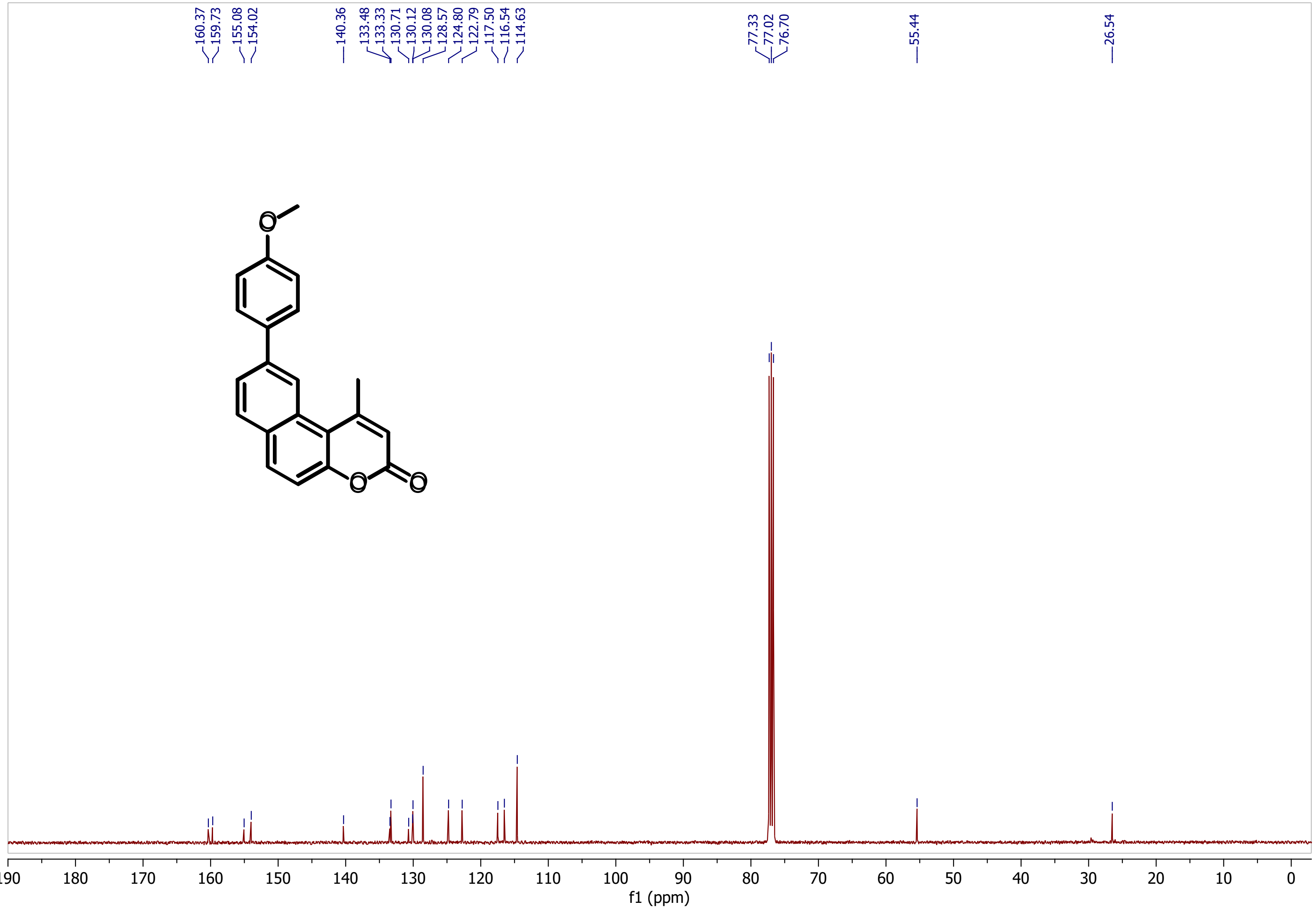

^{1}H NMR of 4fh

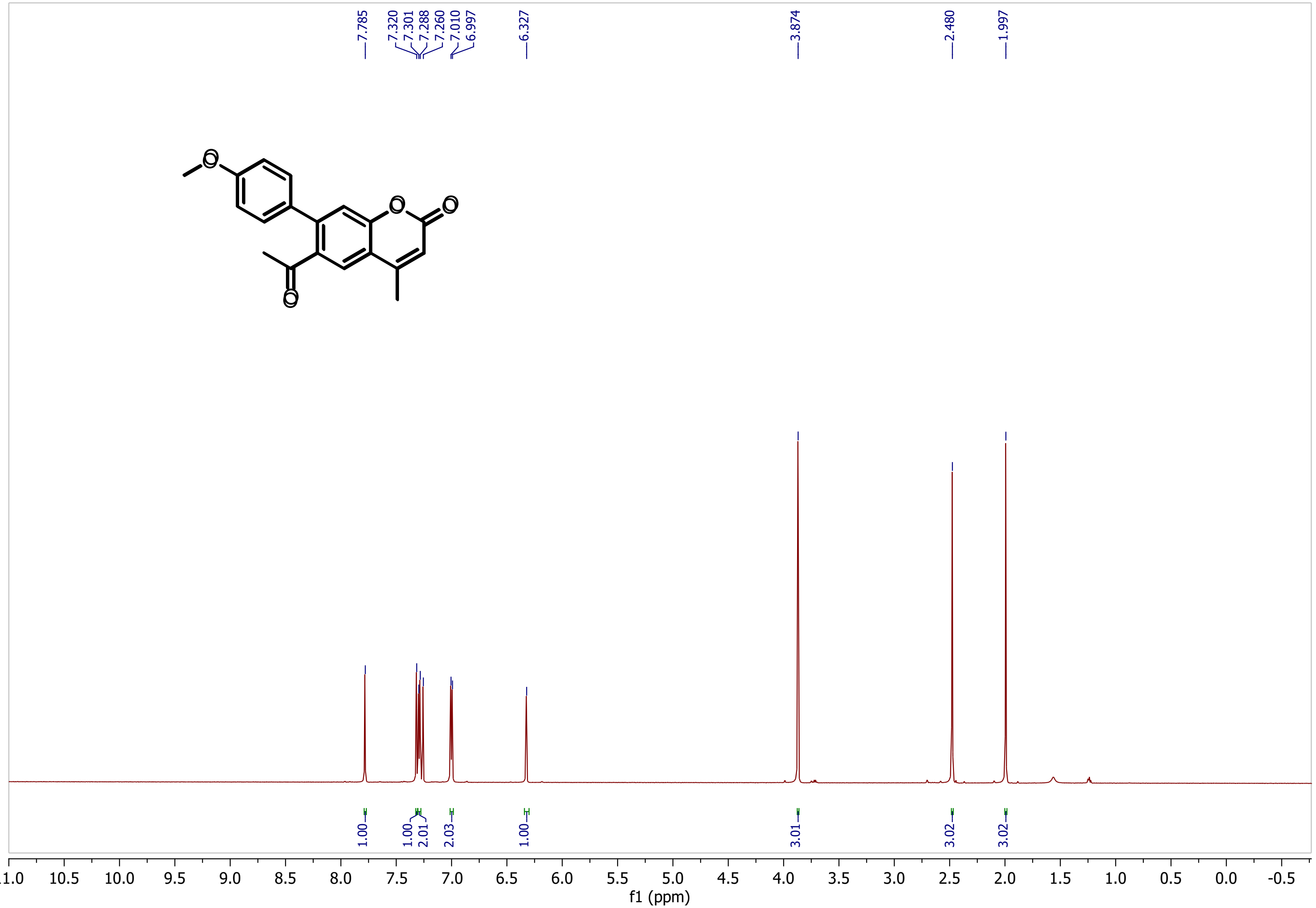
7.785
7.320
7.301
7.288
7.260
7.010
6.997
6.327
3.874
2.480
1.997
1.00
1.00
2.01
2.03
1.00
3.01
3.02
3.02
11.0 10.5 10.0 9.5 9.0 8.5 8.0 7.5 7.0 6.5 6.0 5.5 5.0 4.5 4.0 3.5 3.0 2.5 2.0 1.5 1.0 0.5 0.0 -0.5
f1 (ppm)

^{13}C NMR of 4fh

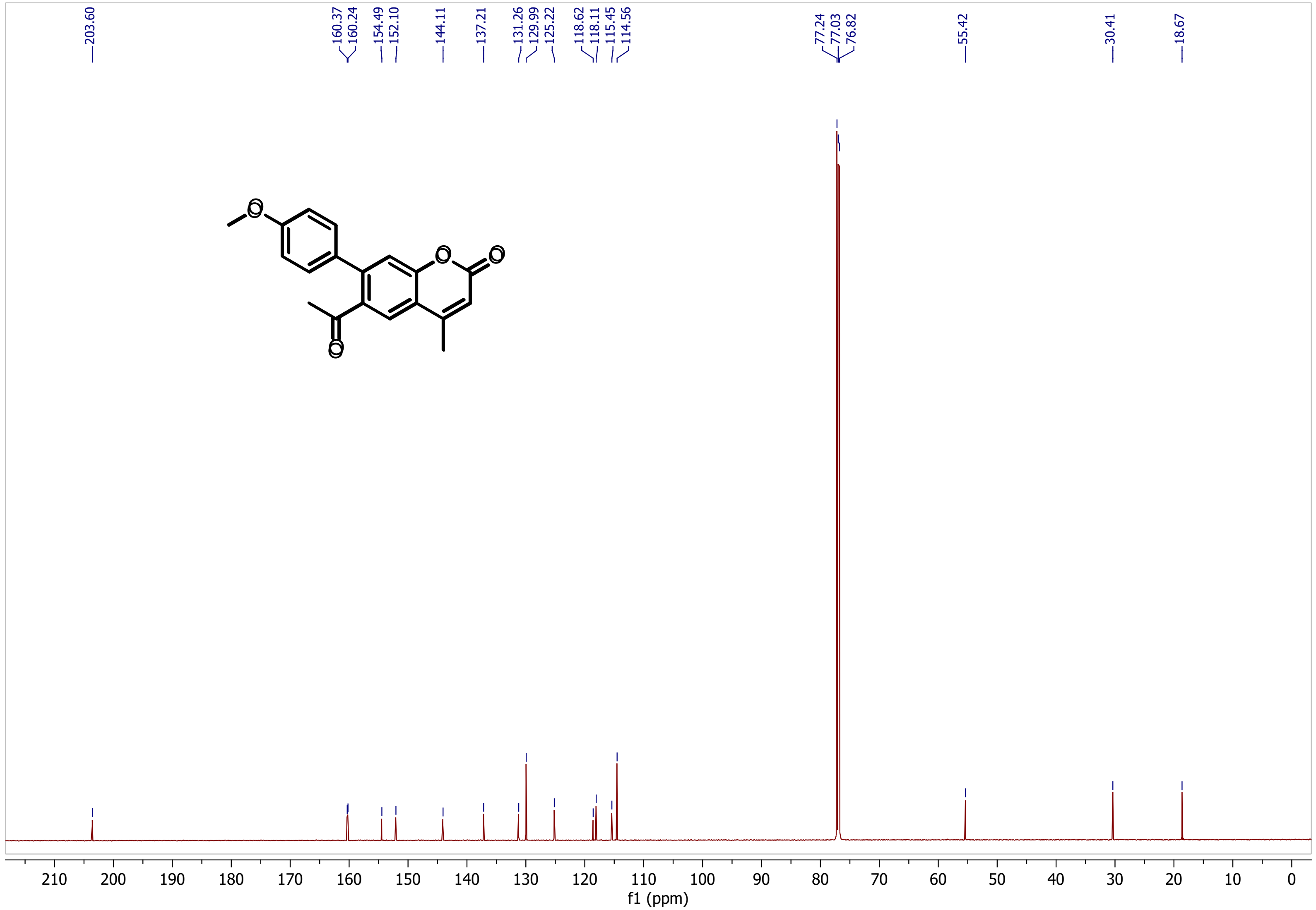

^{1}H NMR of 4gh

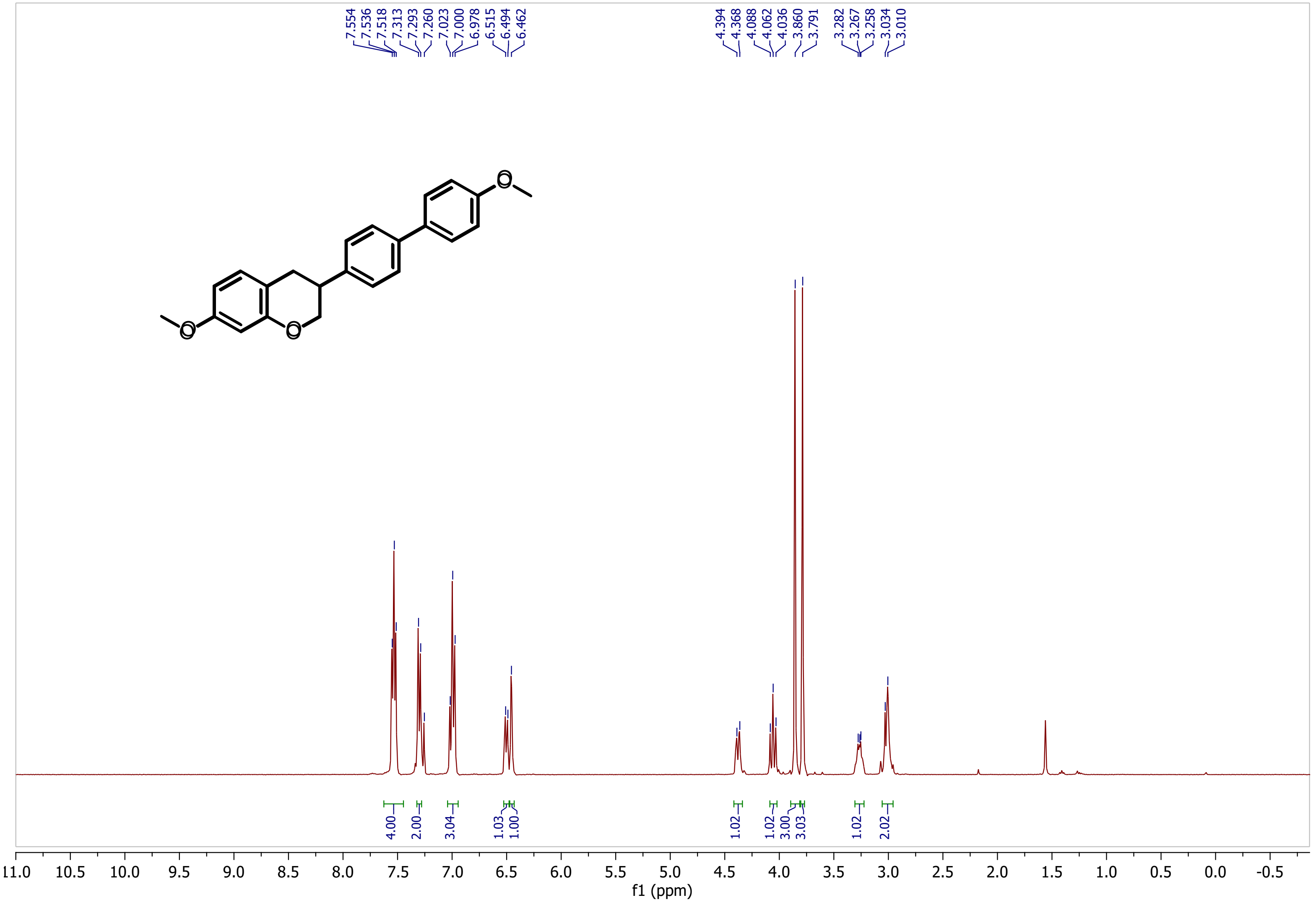
7.554
7.536
7.518
7.313
7.293
7.260
7.023
7.000
6.978
6.515
6.494
6.462
4.394
4.368
4.088
4.062
4.036
3.860
3.791
3.282
3.267
3.258
3.034
3.010
4.00
2.00
3.04
1.03
1.00
1.02
1.02
3.00
3.03
1.02
2.02
11.0 10.5 10.0 9.5 9.0 8.5 8.0 7.5 7.0 6.5 6.0 5.5 5.0 4.5 4.0 3.5 3.0 2.5 2.0 1.5 1.0 0.5 0.0 -0.5
f1 (ppm)

^{13}C NMR of 4gh

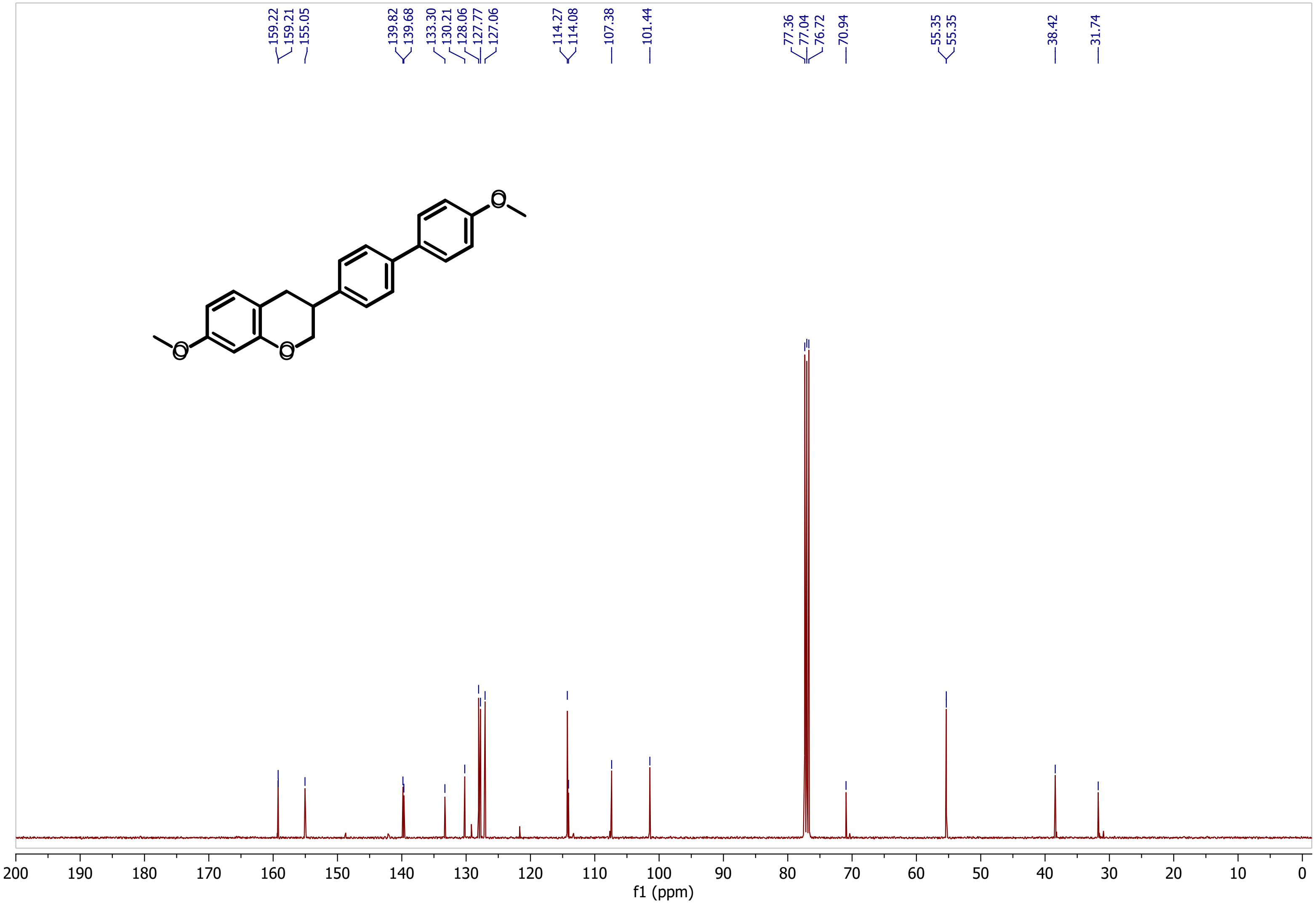

^{1}H NMR of 4hh

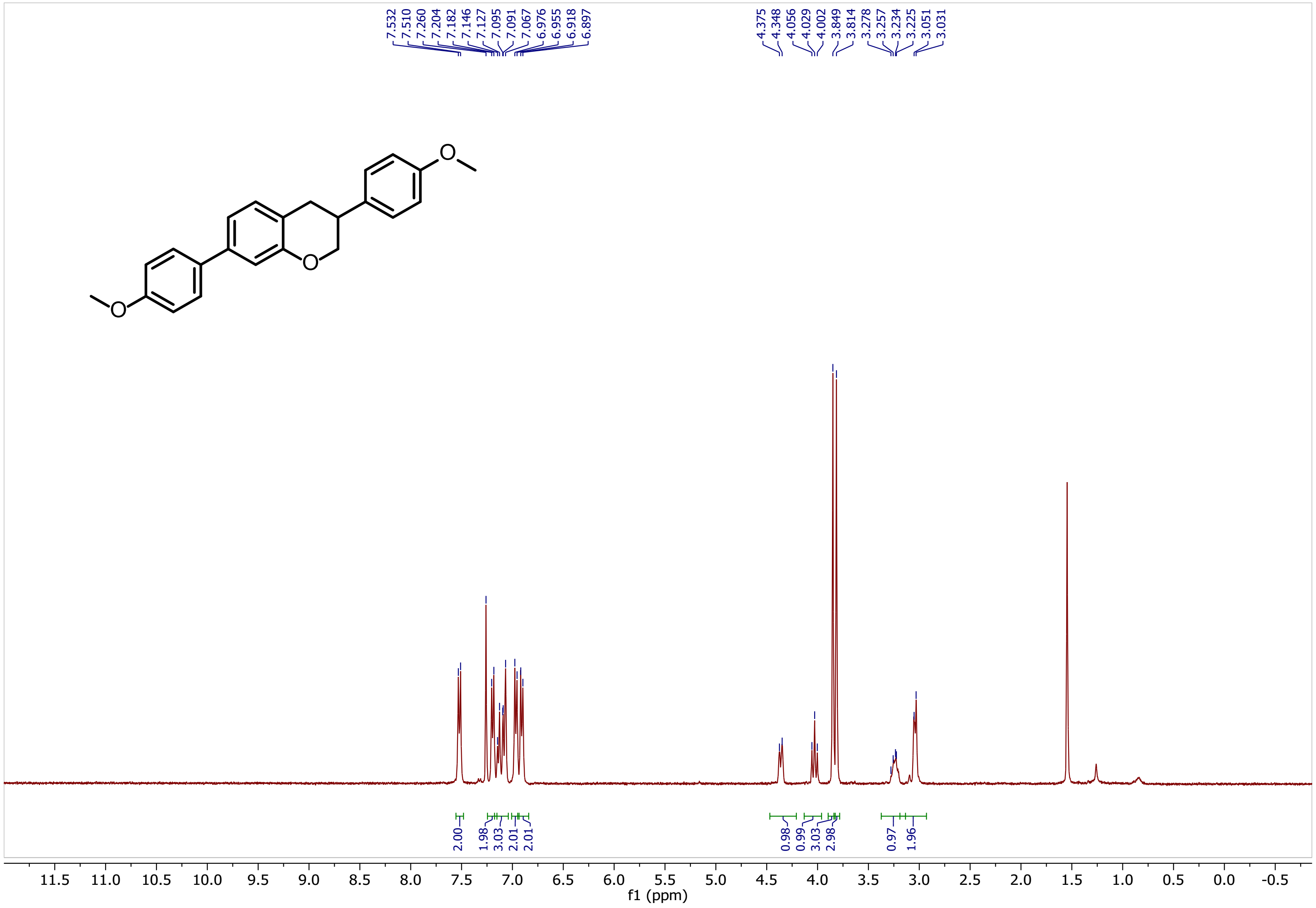

7.532
7.510
7.260
7.204
7.182
7.146
7.127
7.095
7.091
7.067
6.976
6.955
6.918
6.897
4.375
4.348
4.056
4.029
4.002
3.849
3.814
3.278
3.257
3.234
3.225
3.051
3.031
2.00
1.98
3.03
2.01
2.01
0.98
0.99
3.03
2.98
0.97
1.96
11.5 11.0 10.5 10.0 9.5 9.0 8.5 8.0 7.5 7.0 6.5 6.0 5.5 5.0 4.5 4.0 3.5 3.0 2.5 2.0 1.5 1.0 0.5 0.0 -0.5
f1 (ppm)

^{13}C NMR of 4hh

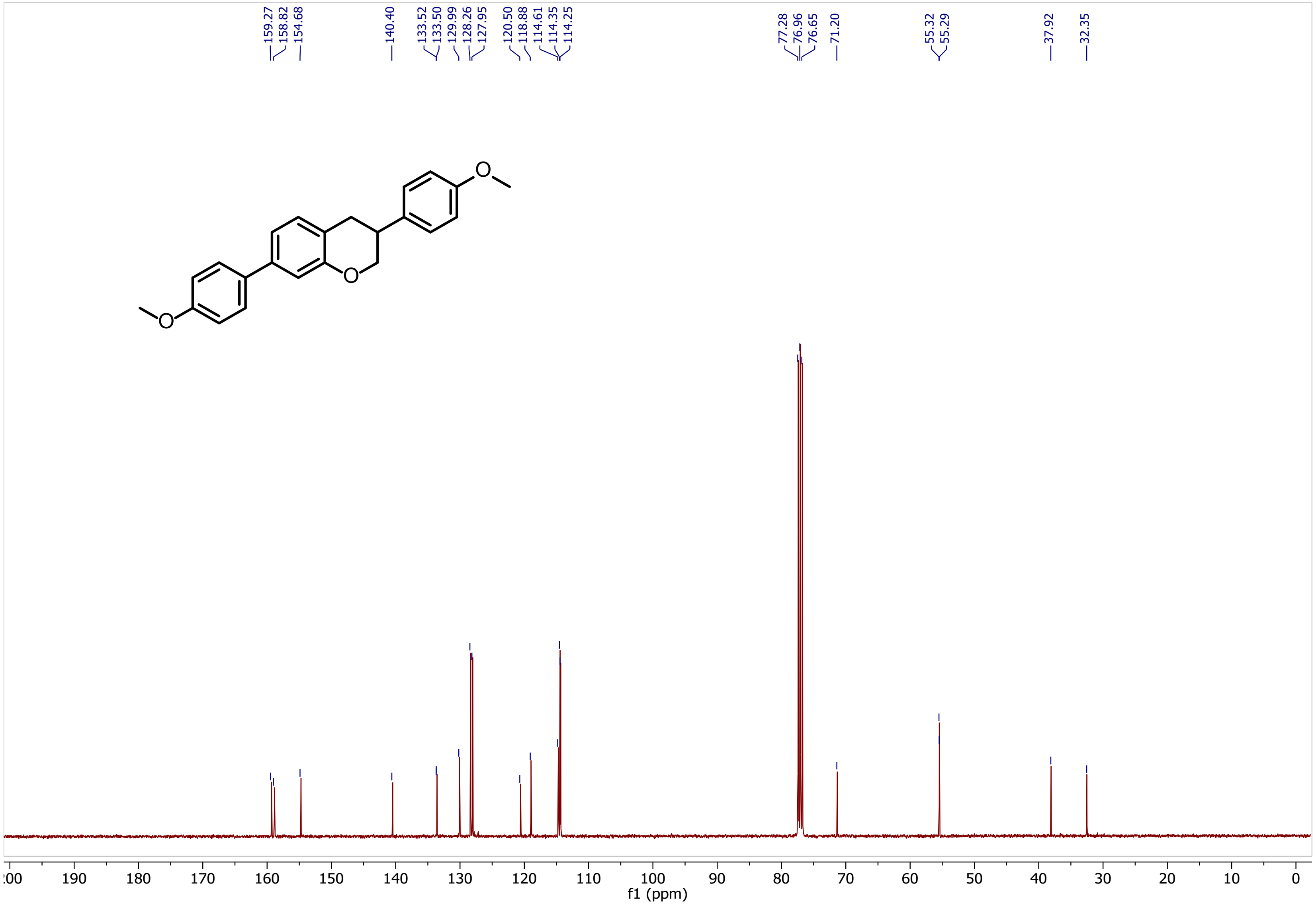

^{1}H NMR of 4ih

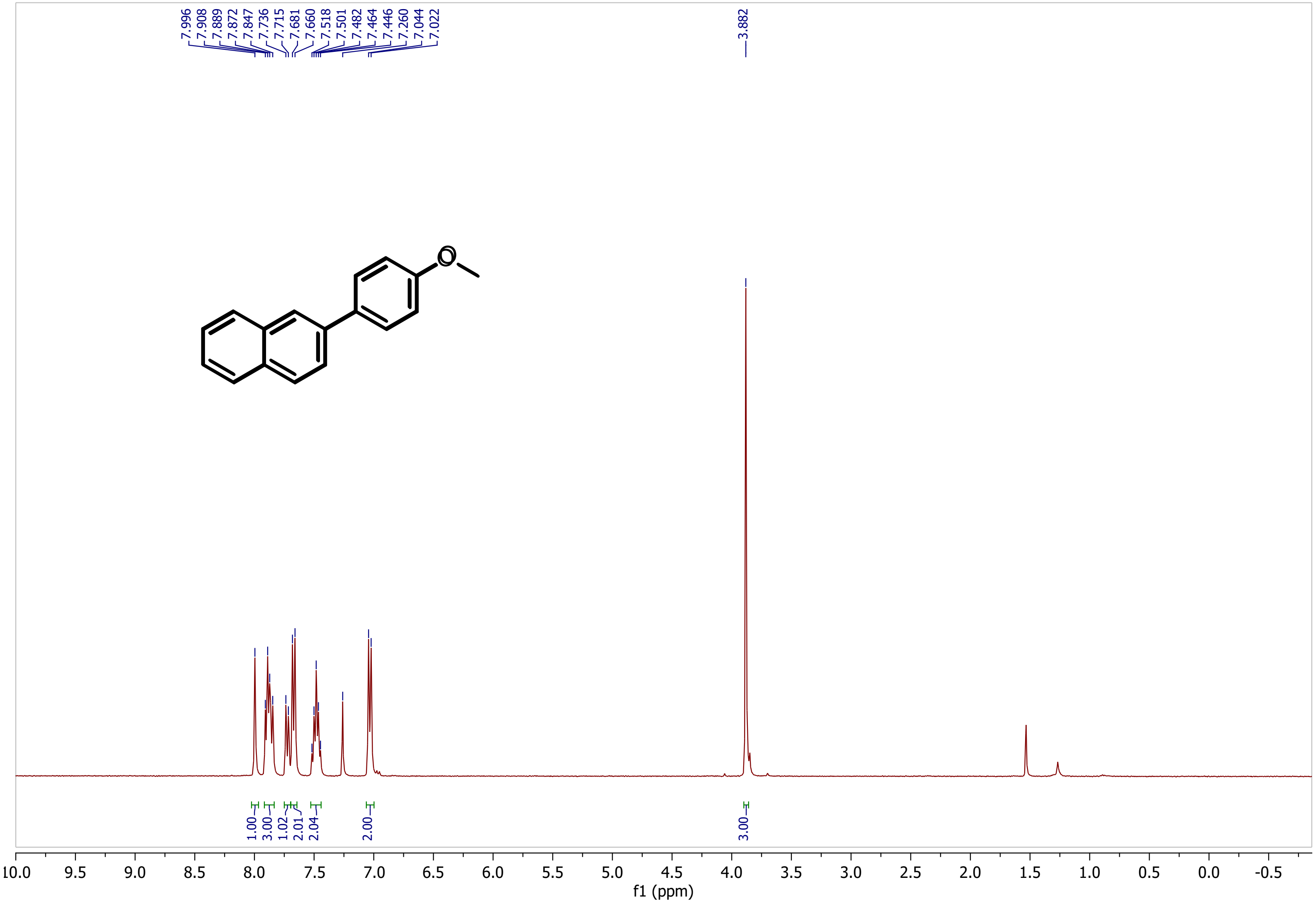
7.996
7.908
7.889
7.872
7.847
7.736
7.715
7.681
7.660
7.518
7.501
7.482
7.464
7.446
7.260
7.044
7.022
3.882
1.00
3.00
1.02
2.01
2.04
2.00
3.00
10.0 9.5 9.0 8.5 8.0 7.5 7.0 6.5 6.0 5.5 5.0 4.5 4.0 3.5 3.0 2.5 2.0 1.5 1.0 0.5 0.0 -0.5
f1 (ppm)

^{13}C NMR of 4ih

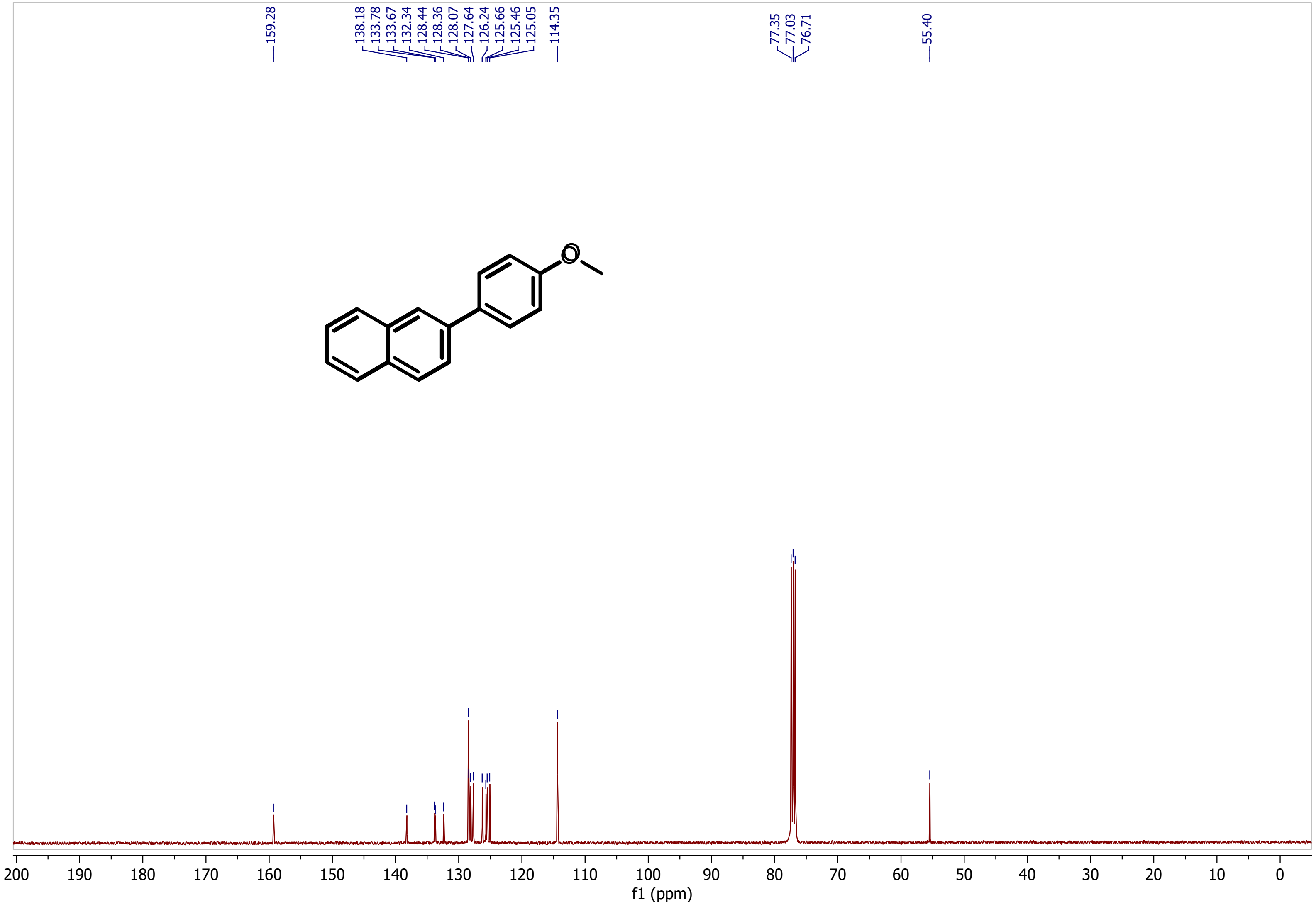

Supplement: Supplementary file 1 [file antioxidants-13-01198-s001.zip › antioxidants-3200303-supplementary.pdf]
